# Type 2 diabetes risk alleles in peptidyl-glycine alpha-amidating monooxygenase influence GLP-1 levels and response to GLP-1 receptor agonists

**DOI:** 10.1186/s13073-026-01630-0

**Published:** 2026-03-29

**Authors:** Mahesh M. Umapathysivam, Elisa Araldi, Benoit Hastoy, Adem Y. Dawed, Hasan Vatandaslar, Johanna E. Mayrhofer, Peter Lindquist, Pamuditha N. Silva, Algera Goga, Geraldine O. Trüllinger, Svenja Godbersen, Shahana Sengupta, Adrian Kaufmann, Søren Krogsgaard Thomsen, Bolette Hartmann, Yi-Chun Chen, Anna E. Jonsson, Hasan Kabakci, Swaraj Thaman, Niels Grarup, Christian T. Have, Lindsay P. Pallo, Kristine Faerch, Anette P. Gjesing, Sameena Nawaz, Jane Cheeseman, Matthew J. Neville, Oluf Pedersen, Mark Walker, Han Sun, Christopher Jennison, Andrew T. Hattersley, Jens F. Rehfeld, Rury R. Holman, Bruce C. Verchere, Torben Hansen, Fredrik Karpe, Jens J. Holst, Mette M. Rosenkilde, Angus G. Jones, Michael Ristow, Mark I. McCarthy, Ewan R. Pearson, Markus Stoffel, Anna L. Gloyn

**Affiliations:** 1 Oxford Centre for Diabetes, Endocrinology & Metabolism, University of Oxford, Oxford, UK; 2 Department of Endocrinology, Queen Elizabeth Hospital, SA Health, Woodville South, Australia; 3 Southern Adelaide and Diabetes and Endocrinology Service, Bedford Park, Australia; 4 Centre of Research Excellence: Translating Nutritional Science to Good Health, Adelaide University, Adelaide, South Australia Australia; 5 Institute of Molecular Health Sciences, Department of Biology, ETH Zurich, Zürich, Switzerland; 6 Institute of Translational Medicine, Department of Health Sciences and Technology, ETH Zurich, Zürich, Switzerland; 7 Department of Medicine and Surgery, University of Parma, Parma, Italy; 8 Division of Population Health & Genomics, School of Medicine, University of Dundee, Dundee, UK; 9 Department of Biomedical Sciences, Faculty of Health and Medical Sciences, University, Copenhagen, Denmark; 10 Departments of Surgery and Pathology and Laboratory Medicine, Centre for Molecular Medicine and Therapeutics, Faculty of Medicine, University of British Columbia, and BC Children’s Hospital Research Institute, Vancouver, BC Canada; 11 Novo Nordisk Foundation Center for Basic Metabolic Research, University of Copenhagen, Copenhagen, Denmark; 12 Copenhagen University Hospital - Steno Diabetes Center Copenhagen, Herlev, Denmark; 13 Division of Endocrinology, Department of Pediatrics, Stanford School of Medicine, Stanford, USA; 14 Oxford Biomedical Research Centre, National Institute of Health Research, Churchill Hospital, Headington, Oxford, UK; 15 Translational and Clinical Research Institute, Newcastle University, Newcastle upon Tyne, UK; 16 Department of Mathematics, University of Bath, Bath, UK; 17 University of Exeter College of Medicine & Health, Exeter, UK; 18 Department of Clinical Biochemistry, Rigshospitalet, University of Copenhagen, Copenhagen, Denmark; 19 Wellcome Centre for Human Genetics, University of Oxford, Oxford, UK; 20 Medical Faculty, University of Zürich, Zürich, Switzerland; 21 Stanford Diabetes Research Centre, Stanford, USA; 22 Present Address: Department of Genetics, Novo Nordisk Research Centre Oxford, Oxford, UK; 23 Present Address: Center for Clinical Metabolic Research, Gentofte University Hospital, Copenhagen, Denmark; 24 Present Address: Novo Nordisk A/S, Bagsværd, Denmark; 25 Present Address: Genentech, 1 DNA Way, South San Francisco, CA 94080 USA; 26 Department of Pediatrics, Division of Endocrinology & Diabetes, Stanford School of Medicine, Centre for Academic Medicine, 453 Quarry Road, Palo Alto, CA 94304 USA

## Abstract

**Background:**

Type 2 diabetes (T2D) is a leading cause of morbidity and mortality worldwide. Despite the availability of multiple glucose-lowering agents, only half of individuals with T2D achieve the recommended HbA1c target of < 7.0%. Precision medicine approaches that leverage patient-specific markers offer a promising strategy to improve therapeutic outcomes. The *PAM* gene encodes the sole enzyme responsible for amidating bioactive hormones, including GLP-1, and harbors two hypomorphic T2D-risk alleles (p.D563G and p.S539W); however, whether PAM regulates GLP-1, a key amidated incretin hormone, and whether this influences response to GLP-1 receptor agonist (GLP-1RA) therapy, remains unknown.

**Methods:**

PAM amidation activity, postprandial GLP-1 levels, and the incretin effect were measured in carriers of *PAM* T2D-risk alleles and matched non-carriers from the Oxford Biobank in a prospective observational study and in Danish cohorts. Inducible whole-body *Pam* knockout mice were generated; gastric emptying was assessed by paracetamol absorption assay with and without exendin-4. Glycemic response to GLP-1RAs was evaluated in a meta-analysis of 1,119 participants across three cohorts (IMI-DIRECT, GoDARTS, PRIBA), with comparative assessment of sulphonylurea, metformin, and DPP-4 inhibitor response.

**Results:**

Carriers of p.S539W and p.D563G alleles demonstrated 52% and 20% reductions in serum PAM amidation activity, respectively. Both human carriers and *Pam* knockout mice exhibited elevated circulating GLP-1 levels; however, p.S539W carriers showed an 18% reduction in endogenous GLP-1 sensitivity. PamKO mice displayed accelerated gastric emptying that was refractory to exendin-4, alongside impaired cAMP signaling downstream of the GLP-1 receptor in the pylorus. In the clinical meta-analysis, p.S539W carriers showed a significantly attenuated HbA1c reduction following GLP-1RA therapy (− 0.69% vs. − 1.24% in non-carriers; *p* = 0.025), representing a 44% relative loss of glycemic benefit; only 11.5% of carriers achieved HbA1c < 7% compared with 25.3% of non-carriers. No differences in response to sulphonylureas, metformin, or DPP-4 inhibitors were observed.

**Conclusions:**

Hypomorphic *PAM* T2D-risk alleles reduce amidating enzyme activity, elevate circulating GLP-1 levels, and impair GLP-1 post-receptor signaling, culminating in a selective and clinically meaningful reduction in GLP-1RA efficacy. These findings establish *PAM* genotype as a novel pharmacogenomic determinant of GLP-1RA response, supporting its incorporation into precision medicine frameworks to optimize drug selection in T2D management.

**Trial registration:**

NCT02723110, NCT02465515 and NCT01144338.

**Supplementary Information:**

The online version contains supplementary material available at 10.1186/s13073-026-01630-0.

## Background

Type 2 diabetes (T2D) affects hundreds of millions of people worldwide and, despite decades of therapeutic advances, continues to be associated with high burden of morbidity and mortality [1]. Current management focuses on reducing glycemic exposure and preventing complications through lifestyle modification and a broad range of pharmacological agents, including metformin, sulphonylureas, DPP-4 inhibitors, SGLT-2 inhibitors, and GLP-1 receptor agonists (GLP-1RAs) [2]. Each drug class acts through distinct mechanisms, yet despite this therapeutic breadth, fewer than half of individuals with T2D achieve the recommended glycemic target of HbA1c < 7.0%. [3, 4]. This gap reflects not only variation in adherence and disease progression, but also substantial inter-individual heterogeneity in drug response that is poorly captured by current prescribing frameworks. Precision medicine, in which patient-specific factors, such as genetic markers, are used to predict therapeutic response offers a compelling strategy to address this challenge and move beyond a trial-and-error approach to drug selection. Central to this opportunity is a better understanding of the molecular pathways that govern glycemic regulation. GLP-1, an incretin hormone secreted postprandially by intestinal L-cells, stimulates insulin secretion, suppresses glucagon, and slows gastric emptying — effects that collectively lower postprandial glucose [5, 6]. Full biological activity of GLP-1, alongside several other glucose-regulating hormones including gastrin and cholecystokinin (CCK), requires C-terminal amidation [7–10]. This post-translational modification is catalysed exclusively by peptidyl-glycine alpha-amidating monooxygenase (PAM), the sole enzyme capable of converting a C-terminal glycine residue to an amide group, a reaction essential for the stability and bioactivity of many hormones [7, 11]. Aside from amidation, PAM also has several non-catalytic functions, including a role in intracellular protein trafficking [12, 13]. Two independent loss of function coding alleles in *PAM* (p.S539W, rs78408340, minor allele frequency (MAF) ~ 1%, OR: 1.47 and p.D563G, rs35658696, MAF ~ 5%, OR: 1.23) increase T2D risk and reduce beta-cell function [14, 15]. We have shown in vitro that PAM deficiency in human pancreatic beta cells causes reduced insulin content and altered dynamics of insulin secretion [12]. These experiments informed on the impact of PAM inactivation on beta cell function in cell autonomous situations but could not assess whether PAM deficiency also contributes to elevated diabetes risk by regulating other hormones affecting insulin secretion, gastric emptying (GE) or other metabolic pathways affected in T2D. *PAM* knockdown in beta cells altered the kinetics of exocytosis and the immediately available pool of insulin granules, which will reduce the effectiveness of GLP-1 in stimulating insulin secretion [12]. As a result, it is likely that in addition to intrinsic effects of PAM loss in the islet, there are additional extrinsic effects through an altered GLP-1 response in carriers of p.D563G and p.S539W [12]. GLP-1 is itself amidated by PAM and interacts with several other amidated peptides that regulate its secretion via multiple mechanisms, which could also influence its efficacy [16, 17]. There is also evidence that genetic variation at this locus contributes to variation in height, waist hip ratio, and BMI supporting a role for *PAM* loss in T2D-risk beyond its direct (intrinsic) effects on beta-cell function [18].

Altered GLP-1 plasma levels or GLP-1 sensitivity in carriers of *PAM* T2D risk alleles could have implications for the efficacy of two commonly prescribed medication classes for T2D: GLP-1RA and dipeptidyl-peptidase 4 inhibitors (DPP-4i). Given that ≈ 10% of individuals carry a loss of function alleles in the *PAM* gene, demonstration of GLP-1 deficit or resistance in carriers of *PAM* T2D risk alleles would impact the medication choice for many individuals with T2D [14, 15]. We hypothesised that *PAM* T2D risk allele carriers would have a reduced incretin effect compared to non-carriers and would have an altered response to GLP-1RA.

## Methods

### Human biochemical and clinical methods

#### Prospective recruit-by-genotype study from Oxford Biobank

Twenty carriers of the low frequency *PAM* p.S539W T2D-risk LoF allele and twenty matched non-carriers (matched for age, BMI and sex) were prospectively recruited to into a double blind, observational, recruit-by-genotype study from the Oxford Biobank (OBB), a biobank of white European individuals with stored DNA without a history of diabetes [19]. Participants were eligible for recruitment if they were male or female, aged 30 to 70 years, able to provide informed consent and had attended an OBB screening visit. Participants were excluded if they had: any other major ongoing disease (cardiovascular, gastrointestinal, metabolic, cancer, psychiatric) that would interfere with the interpretation of the metabolic testing protocols, significant previous gut surgery including gastric banding and significant bowel resection, were involved in another research project that is deemed to add unacceptable levels of variation into glucose and insulin handling or a regular blood donor.

The primary outcome was to assess the impact of *PAM* genotype on the magnitude of the incretin effect as measured by isoglycemic clamp study. Secondary outcomes were to assess the impact of *PAM* genotype on PAM enzyme activity, GLP-1 concentrations, insulin and glucose concentrations during an OGTT. Further exploratory outcomes include postprandial profiles for glucagon, glucose-dependent insulinotropic polypeptide (GIP), gastrin (gly and amide), CCK (gly and amide) and IAPP (gly and amide).

Genotype was reconfirmed on day 1 of the study (Table 1). One pair was excluded due to genotyping error. Subjects underwent a 4 h frequently sampled OGTT and matched isoglycemic clamp (the gold standard assessment of the incretin effect) (Additional file 1: Supplementary methods Sect.  4.1) on separate study days [20]. The effect of genotype on the various outcomes was assessed using a 2-sided t-test, RM-ANOVA or mixed effect analysis as appropriate. All data are displayed as mean ± SD.

**Table 1:** Clinical & biochemical characteristics of genotype-based recall study

	Non-carrier	*p*.S539W carrier	*P* Value
Age (years)	50.9 ± 5.8	50.6 ± 5.9	0.23
Sex (m/f)	13/6	13/6	1.0
BMI (kg/m2)	24.9 ± 3.3	24.4 ± 3.1	0.18
Waist : hip ratio	0.86 ± 0.1	0.87 ± 0.09	0.83
Glucose AUC (mmol.l-1.min-1)	1560 ± 226	1652 ± 301	0.23
Insulin AUC	8422 ± 3582	11,530 ± 7769	0.09
Total GLP-1 AUC (min.pmol/l)	6887 ± 230	7692 ± 304	0.04
Incretin effect (%)	50.7 ± 11.2	47.5 ± 14.4	0.50
Gastrin amide fold change	1.63 ± 0.43	1.47 ± 0.48	0.09
Gastrin gly fold change	1.06 ± 0.18	1.04 ± 0.49	0.71
CCK amide	3.12 ± 4.72	3.58 ± 4.34	0.78
TSH	1.54 ± 0.49	1.34 ± 0.53	0.26
GIP AUC (min.pmol/l)	11,781 ± 4725	11,670 ± 5518	0.95

Data are presented as the mean ± SD. Fold change for gastrin was calculated as the ratio between t_0_ and t_30_. Fold change for CCK amide was calculated at t_0_ and t_60_

### OGTT and isoglycemic clamp

Carriers and matched non-carriers of p.S539W underwent an oral glucose tolerance test (OGTT) and isoglycemic clamp on separate days. The two study days were separated by no less than 7 days. On Study Day 1, participants attended the Clinical Research Unit following a 12 h fast. A retrograde cannula was inserted into the dorsum of the hand for blood sampling. The hand was placed in a “hotbox” to arterialize venous blood. A 75 g oral glucose load was administered over 5 min. Blood was sampled every 5 min for 240 min to determine blood glucose concentration.

On Study Day 2, participants attended the Clinical Research Unit following a 12 h fast. A retrograde cannula was inserted into the dorsum of the hand for blood sampling. The hand was again placed in a “hotbox” to arterialize the venous blood. An anterograde cannula was placed in the antecubital fossa of the contralateral to allow intravenous glucose delivery. A variable intravenous infusion of 20% glucose was administered over 240 min to reproduce the glucose profile of the OGTT.

At 10 time points on both study days samples were drawn (15 ml) and stored in 3 different tube types: potassium EDTA tubes with dipeptidyl peptidase IV (DPP- IV) inhibitor, lithium-heparin tubes and serum tubes. Potassium EDTA and lithium-heparin tubes were placed on ice and immediately centrifuged, then aliquoted on ice, and stored at -80 °C. The serum tubes were left at room temperature for approximately 30 min to facilitate clotting before centrifugation and storage at -80 °C. Amidated GLP-1, non-amidated GLP-1, glucagon and GIP were measured at all timepoints during OGTT. Insulin and glucose were measured in both OGTT and matched isoglycemic clamp.

The study utilized an adaptive study design with an interim analysis at 40 volunteers (20 v 20) with the possibility of adding an additional 20 volunteers to the study (10 v 10) if the criteria for futility or clear effect are not met. The criteria were: stop and reject null hypothesis if t > 2.490 and stop and accept null hypothesis if t < 1.033. If the t fell between these values an additional 20 volunteers (10 v10) were to be recruited. The decision to stop or include additional volunteers was be based on the incretin effect (primary outcome) and the study plan and full adaptive design are listed on the clinicaltrials.gov registry (NCT02723110). This provided a 90% chance of detecting a difference of 10% with an alpha 5% (estimated SD of 10%) in incretin effect at the first stopping point.

### Retrospective clinical cohorts

**Fig. 1:**
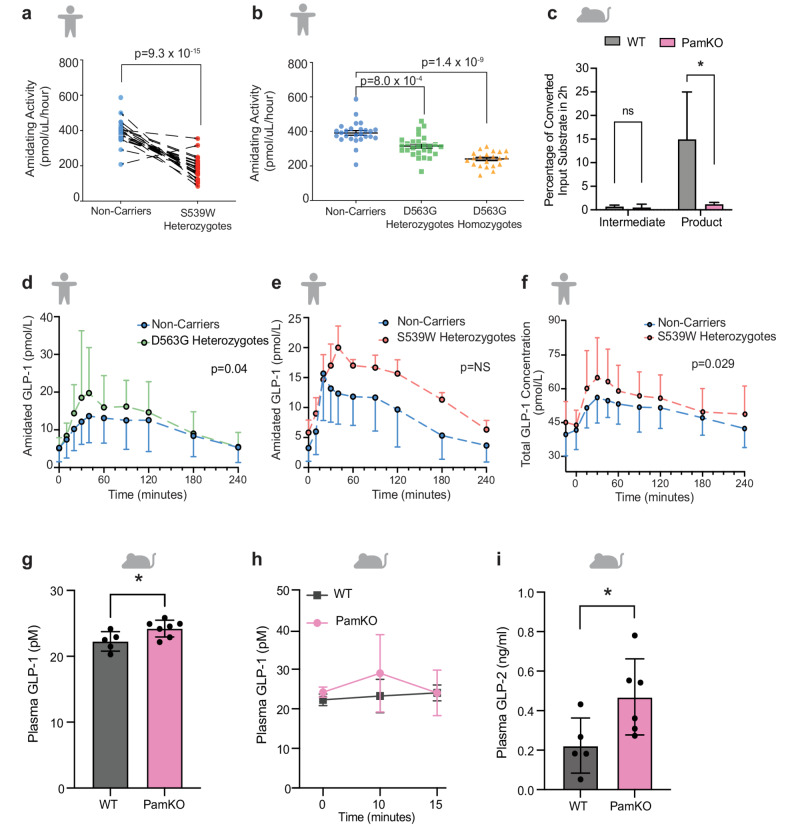
Amidation activity and GLP-1 profiles in carriers of *PAM* loss-of-function alleles and in PamKO compared to WT littermates. **a** Reduction in serum enzyme activity in heterozygous carriers of p.S539W (*N* = 19) compared to age, sex and BMI matched non-carriers (*N* = 19) (left). All samples were measured in triplicate and the data points presented above are the mean of the triplicates. Dashed lines connect the BMI, age and gender matched non-carrier to the corresponding carrier. **b **Reduction observed in heterozygous (*N* = 27) and homozygous carriers (*N* = 21) of p.D563G compared to age, sex and BMI matched non-carriers respectively. Data have been normalised to non-carriers to allow comparison between genotypes. Carriers were 1:1 matched for age, gender, and sex. All samples were measured in triplicate and the data points presented above are the mean of the triplicates. The long black line represents the mean amidating activity +/- the SEM. **c** PAM-mediated conversion of Dansyl-YV-OH-G intermediate and Dansyl-YV-NH_2_ product from Dansyl-YVG (in %) in an amidation assay of pituitary extracts from PamKO and WT littermate control mice after 3 h from the start of the assay (*n* = 2,2). Error bar represents SD. **d**, **e**, Amidated GLP-1 profile of carriers of p.D563G (**d**) and p.S539W (**e**) and age, gender and BMI matched non-carriers following a 75 g OGTT in the Family Study. Each data point is the mean amidated GLP-1 concentration ± SD. Panel a demonstrates the amidated GLP-1 profiles of 24 carriers of p.D563G and 48 matched non-carriers. Panel a demonstrates the amidated GLP-1 profiles of 3 carriers of p.S539W and 6 non-carriers. **f** Total GLP-1 (sum of amidated and non-amidated GLP-1) plasma profiles of 19 heterozygous carriers of S539W and 19 age, gender and BMI matched non-carriers in the prospectively performed 75 g OGTT. Each data point is the total GLP-1 concentration ± SD. Analysis was performed using RM-ANOVA. Note: total GLP-1 is displayed as amidated and non-amidated GLP-1 was measured but there was no difference in the ratio between amidated and non-amidated GLP-1 at any time point and both forms of GLP-1 have equal biological activity [16]. **g** Plasma GLP-1 levels of fasted PamKO mice compared to WT littermates (*n* = 5,7). **h** Plasma GLP-1 levels at indicated timepoints during an oGTT in PamKO compared to WT littermates (*n* = 5,7). **i** Plasma GLP-2 levels of fasted PamKO mice compared to WT littermates (*n* = 5,6). Data are presented as mean ± SD; two tailed *t* test (**d**,** f**) and 2-way repeated measures ANOVA with Sidak’s multiple comparisons test (**e**), *P < 0.05

Retrospective analysis of intact GLP-1 (7–36 amide and 9–36 amide) profiles following a 75 g OGTT was performed in two Danish cohort studies (AdditionPro and Family Study) [21, 22]. Full details of the cohorts is available in primary publications [21, 22]. Demographics of the 2 studies are provided in table S1 (Additional file 1: Table S1). Normoglycemic carriers of the T2D -risk alleles p.S539W and p.D563G were matched for age, BMI and sex to two non-carriers. Individuals with T2D were excluded prior to analysis to minimize effects associated with treatment and avoid reduction in incretin effect observed in overt hyperglycaemia [23]. In the Family Study, amidated plasma GLP-1 levels (7–36 amide and 9–36 amide) were available at 10 timepoints following an OGTT. We compared GLP-1 profiles and the AUC_120_ in 26 Danish carriers of the p.D563G allele and 56 matched non-carriers (Fig. 1d) [19, 20]. In the Addition-PRO GLP-1 levels were available at 3 time points. Heterozygous carriers of p.S539W and p.D536G alleles were matched 1:2 to non-carriers (14 vs. 7 and 290 vs. 145 respectively).

We prospectively measured GLP-1 7–36 amide and GLP-1 7–37 Gly in the stored plasma of 76 heterozygous carriers of p.D539W and 70 non-carriers matched for age, BMI and sex from the OBB. Details of the full cohort are available from the primary publication [19].

### Biochemical measurement

To establish the impact of T2D associated LoF alleles (p. D563G & p.S539W) on PAM enzyme activity in carriers, stored serum samples from the OBB and a prospective collected serum from a recruit-by-genotype study were analyzed. PAM activity was measured using a previously described radiotracer method (Additional file 1: Supplementary methods) in 24 heterozygous carriers of p.S539W, 27 heterozygous carriers of p.D563G and 21 homozygous carriers of p.D563G and age, sex and BMI matched non-carriers [24, 25]. All individuals were normoglycemic and of white European background and measurements were performed in triplicate.

Plasma glucose was measured using the ilab 650 Analyser (Instrumentation Laboratory Ltd, Warrington, UK) as previously described [19]. Serum insulin concentrations were measured using the Human Specific Insulin RIA Kit (EMD Milipore, Billerica, USA) [19]. Radioimmunological determinations of intact, amidated and glycine extended plasma GLP-1 concentration were performed as described previously [26]. Gastrin and CCK were measured using non-commercial antibody-based assays as previously described [27].

Serum levels of human amidated islet amyloid polypeptide (IAPP) and glycine-extended (non-amidated) IAPP levels were measured in a blinded manner using in-house developed Meso Scale Discovery Electroluminescence ELISAs (Rockville, MD, USA) as previously described [28]. In brief, both assays utilized a monoclonal capture antibody (F002; binds all molecular forms of (pro)IAPP) and a monoclonal detection antibody (F025; binds the C-terminally amidated region of IAPP, and F084; binds the glycine extended, non-amidated IAPP C-terminus). Antibodies were provided by MedImmune (Gaithersberg, MD, USA).

### Pharmacogenetics cohorts and methods

Response to GLP-1RA and other oral hypoglycemic agents (DPP-4i, metformin and sulfonylurea) were compared between carriers and non-carriers of *PAM* p.S539W and p.D563G carriers. Response to GLP-1RA was initially assessed by comparing the response to treatment of 1,119 participants with T2D treated with GLP-1RA in a meta-analysis of the three similar cohorts Innovative Medicines Initiative – DIabetes REsearCH on patient straTification (IMI-DIRECT) [29], Genetics of Diabetes Audit and Research in Tayside Scotland (GoDARTS) [30] and Predicting Response to Incretin Based Agents (PRIBA) [31] and replication was sought in the methodologically different GSK-HARMONY Trial [32] and the EXSCEL Trial [33]. Cohort details, including sample size of each cohort, allele frequency in the population are provided in supplemental information (Additional file 1: Supplementary methods Sect.  3.1–3.5). In the discovery meta-analysis, response was determined in all 3 studies by HbA1c change from baseline (day of initiation of medication) to the HbA1c at 6 months of therapy. The clinical model for assessing treatment response was developed using linear regression and backward elimination through the stepAIC function in the MASS package in R. A linear regression was performed adjusting for clinical covariates: baseline HbA1c, age at diagnosis, duration of diabetes, number of oral glycemic agents (OHA) at initiation of GLP-1RA, insulin dose and change in OHA. A meta-analysis was performed using a fixed effects model in the Forrest package of R.

Exploratory analysis was conducted in two clinical trials. Both trials were prospectively registered (NCT02465515 and NCT01144338). In the GSK-Harmony trial data (*N* = 1,292), the effect of genotype on HbA1c was determined using a similar model. The method differed due to differences in study design which include that the change in HbA1c was calculated from measurements taken at baseline and at 8 months. Significantly, in the GSK-Harmony trial there was the opportunity for a dose increase at 5 weeks if it was felt that participants were not responding. The study was conducted over multiple sites so in addition to the model used in the discovery meta-analysis study “site” was included as a factor in the clinical model.

In the EXSCEL trial, there was limited availability of clinical co-variates due to data sharing restrictions. The full model of available co-variates included baseline HbA1c, sex, BMI and duration of diabetes. Analysis suggested no additional improvement in the model with the addition of sex, BMI and duration of diabetes therefore the reduced model using only baseline HbA1c as a covariable is presented. Glycemic response was defined as HbA1c change at 6 months following initiation of GLP-1RA. Only a subset of the data were available to investigators due to data sharing restrictions (*N* = 1506). Details of cohorts for these analyses are provided in supplemental information (Additional file 1: Supplementary methods Sect.  3.1). Finally, to examine the effect of intermittent or continuous GLP-1R receptor stimulation, the impact of genotype was characterized for both short and long-acting agonists, data from all 5 studies were pooled (Additional file 2: Fig. S8). The effect of *PAM* genotype on metformin and sulphonylurea response was also assessed in GoDARTS and IMI-DIRECT and used the same model as the assessment of GLP-1 in these cohorts.

### In vitro studies

#### Insulin secretion in the human EndoC-βH1 cell line

EndoC-βH1 cells were cultured, platted in a 96-well plate and transfected with siRNA as described previously or incubated for 48 h with either DMSO or 500µM 4-Phenyl-3-butenoic acid (PBA, Sigma #155322) [34]. Platted cells were incubated the night prior to the experiment in culture medium with 2.8 mM glucose. On the day of the assay, cells were incubated in glucose-free culture medium for 1 h and then stimulated with either 1 mM or 10 mM glucose. The latter stimulation was complemented with either GLP-1 (7–36) amide (1 nM, BACHEM #4030663), tolbutamide (200 µM, Fluka Analytical #T0891), or Forskolin (10 µM, Merck #F3917). Residual cells were removed by centrifuging the collected supernatants (4 °C, 700 g, 5 min) and 50µL of the supernatant was stored at -20 °C until the assay. Samples to measure insulin content were harvested in 100µL of acid ethanol (1.5% conc. HCl, 75% ethanol, and 23.5% distilled H_2_O) in the 96-well plate and stored at -20 °C. Insulin concentrations of both supernatants and cellular contents were determined using Insulin (human) AlphaLISA Detection Kit (PerkinElmer). Data presented are the result of 3 biologically independent experiments on two different passages of EndoC-βH1 lines. Each biological replicate is the average of 3 technical replicates. We used a 2-way ANOVA followed by a Tukey post hoc test in GraphPad to evaluate the interaction between the transfection (siControl vs. siPAM) or treatment (DMSO vs. PBA), and the various stimulations of insulin secretion detailed above.

### Animal studies

#### Generation & characterization of Pam knockout mouse models

All animal experiments were in accordance with institutional guidelines and approved by the kantonale Veterinäramt Zürich.

Animals were housed in a pathogen-free animal facility at the Institute of Molecular Health Sciences at ETH Zürich. Mice were maintained in a temperature- and humidity-controlled room on a 12 h light/ dark cycle (lights on from 6:00 to 18:00). Mice were given ad libitum access to a standard laboratory chow and water. All animals were at least 8 weeks of age. Experiments were performed independently in both sexes, and figures display representative experiments. *Pam*^*fl/fl*^ mice were generated in the ETHZ EPIC facility by injecting binocysts with Pam-targeted ES cells obtained from EUMMCR (clone EPD0607_1_A11). Founders (tm1a – Pamflneo/flneo) were screened for the presence of the targeted allele and neo cassette, and bred with FLP-Deleter (B6.129S4-Gt(ROSA)26Sortm1(FLP1) Dym/RainJ) to remove the neo cassette (to obtain tm1c – *Pam*^*fl/fl*^ allele) (Additional file 2: Fig. S1A). UBC-Cre mice (Tg[UBC-cre/ERT2]1Ejb - purchased from Jackson Laboratories) were crossed with *Pam*^*fl/fl*^ to obtain *UBC-Cre PAM*^*fl/fl*^. To create PDX1-Cre *Pam*^*fl/fl*^ mice, B6.FVB-Tg(Pdx1-cre)6Tuv/J were crossed with *Pam*^*fl/fl*^ mice.

#### Tamoxifen injection for Cre-mediated Pam^fl/fl^ allele recombination

Mice at 4/5 weeks of age were administered daily intraperitoneal injections of 2 mg tamoxifen (T5648, Sigma) for 5 days, dissolved at a concentration of 20 mg/mL in 10% Ethanol/90% corn oil.

#### Oral glucose tolerance test

Mice were fasted for 6 h and D-glucose (Sigma, 49139) solution (2 g/kg) administered by gavage. Blood glucose values were measured by tail nick with a Bayer Contour XT glucometer at 0, 15, 30, 45, 60, and 120 min after injection.

#### GLP-1 measurement

Mice were injected intraperitoneally with dipeptidyl peptidase-4 (DPP-4) inhibitor Sitagliptin (Merck, 3 mg/kg) at *t* = 0 and after 30 min, D-glucose (Sigma Aldrich 2 g/kg) was administered orally by gavage. Blood was sampled 5 min thereafter and added to 5 µl Aprotinin (Sigma, 5 mg/ml), 2 µl EDTA (0.5 M) and, 3 µl DPP-4 Inhibitor (Millipore) on ice. Blood was collected from the tail vein, serum isolated and GLP-1 content was measured with GLP-1 ELISA (Merck).

#### Paracetamol gastric emptying assays

10 mg/mL of paracetamol (Acetaminophen, Panadol) and 0.2 g/mL glucose in PBS were administered by gavage (at a final dose of 0.1 mg/g and 2 mg/g of body weight). Blood was collected from tail vein before gavage, at 15, 30, 45 and 60 min after gavage, and concomitantly blood glucose was measured with a Bayer Contour XT glucometer. Serum was used to measure paracetamol at each time point (Paracetamol Test Kit Triple Enzyme, K8003, CLS diagnostics, UK). In assays including Exendin 4 (Exenatide, Bydureon, AstraZeneca), the compound was injected intraperitoneally 30 min prior gavage at the concentrations of 10 nmol/kg.

#### Measurements of tissue cAMP

Mice were injected i.p. with Exenatide (10 nmol/kg) and euthanized after 3 min by cervical dislocation. Tissues were immediately harvested and snap frozen in liquid N_2_. For cAMP measurements, frozen tissues were disrupted in 2 ml Eppendorf tubes containing 5 mm stainless steel tissue lyser beads (Qiagen #69989) in a tissue lyser (TissueLyser II, Qiagen) containing lysis buffer at 30 Hz for 2 min. Suspensions were centrifuged for 10 min at 10,000 x g at 4 °C and supernatants were transferred to a new tube and cAMP was measured according to the manufacturer’s instruction of the cAMP parameter assay kit, a competitive enzyme immunoassay designed to measure cAMP (R&D Systems, #KGE002B). cAMP concentrations were further normalized to weight or protein content as measured by BCA assays.

#### Total intestinal GLP-1 measurements

Mice were sacrificed and 1 cm samples of the distal ileum and colon harvested, thoroughly rinsed in cold PBS and snap-frozen on dry ice and stored at -80 °C until protein extraction. Intestinal tissue segments were homogenized, lysed (1 M Tris-HCl, 5 M NaCl, 1% (v/v) Igepal CA-630, 0.5% (w/v) sodium deoxycholate monohydrate, and one tablet of EDTA-free protease inhibitor cocktail), and centrifuged at 10,000 x g for 10 min to prepare the tissue extracts. GLP-1 levels tissue extracts were measured using an active GLP-1 mouse ELISA (Cystal Chem, #81508) with 100% cross-reactivity to GLP-1 (7–36 amide, GLP-1 (9–36) amide, GLP-1 (1–36) amide). The quantification of GLP-1 levels in tissue extracts was normalized to the protein levels of tissue lysates, which were determined using a BCA Protein Assay Kit (Thermo Fisher Scientific, #A55860).

#### Pancreatic islet isolation

Islet isolation was performed according to a modified protocol of Zmuda et al., 2011 [35]. To perfuse the whole pancreas the Ampulla of Vateri was clamped and 2 mL of Liberase (2.5 mg/ml) (Sigma 05401127001), diluted in RPMI 1640 (Sigma R7509-6 × 500ML), was injected through the common bile duct. The pancreas was excised and digested at 37 °C for 18 min. After digestion the pancreas was mechanically sheared by shaking. Islets and exocrine cells were collected by centrifugation and the exocrine cells were separated from the islets by Histopaque-1077 (Sigma, 10771) density gradient (900 x g, 20 min, with slow acceleration / deceleration). The islets were collected with a cell strainer (70 µM) and recovered in RPMI cultivation media supplemented with 10% FBS, 100 U/ml penicillin/streptomycin, 10 mM HEPES pH 7.4, 2 mM Gibco™ GlutaMAX (Thermo Fisher, 35050061), 1 mM Gibco™ Sodium Pyruvate (Thermo Fisher, 11360-039).

#### Ex vivo glucagon secretion and glucose-stimulated insulin secretion (GSIS)

After isolation pancreatic islets were recovered for 1 h in RPMI islet cultivation medium. Islets of each 3 mice per genotype were handpicked and pooled into KRBH (Thermo Fischer, J67795.AP) starvation buffer containing 1 mM glucose, supplemented with 0.1% fat free BSA (Sigma, A8806-5G). The islets were starved for 1 h at 37 °C in 5% CO_2_ atmosphere. For the assays, 20 islets per replicate were handpicked into a well of a non-tissue culture treated 24-well plate containing 1 mM low glucose (LG) secretion buffer (supplemented with 0.1% fat free BSA). The stimulation with 1 mM LG was carried out at 37 °C for 30 min, then the supernatant was collected. The islets were transferred into a 24-well plate containing either KRBH (supplemented with 0.1% fat free BSA) with 11 mM glucose (HG) or KRBH (supplemented with 0.1% fat free BSA) with 11 mM glucose and 20 nM of GLP-1 (HG + GLP-1). The secretion assay was performed at 37 °C, the supernatant was collected after 30 min. Supernatants of LG and HG or HG + GLP-1 were centrifuged for 1 min at 100 x g to remove cells and debris. The clarified supernatant was transferred to a fresh 96-well plate. The islets from each well were handpicked into Acid-Ethanol solution to extract the total insulin. Secreted glucagon and insulin, as well as total glucagon and insulin were measured by Glucagon Insulin Ultra Sensitive ELISA Kit (Alpco, 80-INSRTU-E10-AL).

#### RNA isolation and quantification

TRIzol reagent (Invitrogen, 15596-026) was used for RNA isolation according to the manufacturer’s protocol. RNA was reverse transcribed using High Capacity cDNA Reverse Transcription Kit (Applied Biosystems, 4368813). Quantitative PCR was performed in an LC480 II Lightcycler (Roche) and using gene specific primers and Sybr Fast 2x Universal Master mix (Kapa biosystems, KK4611). qPCR Primers:

Glp1r-F: 5’-ACGGTGTCCCTCTCAGAGAC; Glp1r-R: 5’-ATCAAAGGTCCGGTTGC AGAA; Gcg-F: 5’-GCTTATAATGCTGGTGCAAG; Gcg_R: 5’-GTCCTCATGCGCTTC TGTCT; 36B4-F: 5’-GCCGTGATGCCCAGGGAAGACA; 36B4-R5’-CATCTGCTTGG AGCCCACGTTG. Results were normalized to 36b4 mRNA levels.

#### HPLC-based YVG amidation assay

Custom made Dansyl-YVG peptide, and control amidated product Dansyl-YV-amide, was synthesized by JPT peptide technologies GmbH. The amidation assay method is a slight adaptation of the method described by Eipper and colleagues [10, 25, 36]. Tissues were flash frozen, then amidation buffer added (NaTES pH7 20 mM, Mannitol 10 mM, Triton X-100 1% v/v freshly open at each lysis buffer preparation) additioned with fresh 1 mM pepstatin, 1 mM PMSF, 1 mM soya beans trypsin inhibitor (all from Sigma). Samples were homogenized for 2 min at 30 Hz with TissueLyser II (Qiagen), then underwent three freeze-thaw cycles before removing debris with centrifugation at 2000 rpm for 15 min at 4 °C, and supernatant quantified and stored frozen. 50 mg of tissue lysate was added to obtain a total of 50 µL of the amidation assay buffer composed of: Catalase 100 ug/mL (freshly prepared), 2 mM L-ascorbic acid (freshly prepared), ZnCl_2_ 2 µM, CaCl_2_ 2 µM, CuSO_4_ 75 µM, NAMES 100 mM, pH 5.5, 0.15 mM Dansyl-YVG. All chemicals were from Sigma. The reaction was then incubated for 2, 4–6 h in a 37 °C water bath before being collected and spun down in Amicon^®^ Ultra 0.5 mL filter vial (Merck Millipore). The flow through was then measured via chromatographic analysis of chemically converted Dansyl-YVG into Dansyl-YV-NH2 or Dansyl-YVG(OH) YVG substrate into the Dansyl-YV- hydroxyglycine intermediate and the amidation product dansyl-YV-N2 by means of high-performance liquid chromatography (HPLC) as described by Ul-Hasan and colleagues [36]. The absorbance of substrate, intermediate and product were detected at 220 and 280 nm on a C18 analytical column over a linear gradient ranging from 22% to 25% of solvent (acetonitrile) in 20 min.

### Assessment of GIP response on mouse islets

GIP assessment was performed in mice from the Mains and Eipper lab (RRID: IMSR_JAX:034076), which were crossed with Ins1Cre mice (RRID: IMSR_JAX:026801). After isolation, pancreatic islets were recovered overnight in RPMI islet cultivation medium. Islets from each mouse (Wt, *n* = 6; bPamKO, *n* = 9) were handpicked into triplicates (20 islets per technical replicate) in 1.7 mL Eppendorf tubes and washed once with PBS. KRBH starvation buffer containing 1 mM glucose, supplemented with 0.1% BSA (Sigma, A7888), was added to the islets for 1 h at 37 °C in a 5% CO2 atmosphere. After removal of the starvation buffer, islets were exposed to the secretion buffers for 45 min as follows: 1 mM low glucose (LG) KRBH, 11 mM glucose (HG) KRBH, and 11 mM glucose plus 20 nM GIP (HG + GIP). The supernatant buffer was collected between incubations. Supernatants from LG, HG, and HG + GIP were centrifuged at 100 x g for 5 min to remove cells and debris. The clarified supernatant was transferred to PCR strips. The islet pellets were freeze-thawed and lysed with RIPA buffer to extract the total insulin.

### GLP-1 (7–36) and GLP-1 (7–37) activity assays

#### Cell culture and transfection

The transfections and assays were performed as described by Van der Velden and colleagues [37]. HEK293A cells were grown in Dulbecco´s modified Eagle´s Medium (DMEM) supplemented with 10% fetal bovine serum (FBS), 1% L-glutamine, 180 units/ml penicillin and 45 µg/ml streptomycin in a humidified CO_2_ incubator at 37 °C. The cells (10^6^ cells/T25 flask in supplemented DMEM) were transfected using calcium phosphate transfection for the cAMP and the competition binding experiments by mixing 10 µg hGLP-1R with 120 µl TE buffer and 15 µl CaCl_2_. The DNA mixture was added into 120 µl 2xHBS and incubated for 45 min before adding it dropwise to the cells. The transfection was terminated after 5 h by replacing the transfection medium with 5 ml supplemented DMEM.

For the β-arrestin 2 recruitment, HEK293A cells (750,000 cells/well in supplemented DMEM) were seeded in a transparent 6-well cell culture plate one day before the transfection. The cells were transfected using polyethylenimine (PEI) by mixing 2.34 µg PEI with 182.2 µl non-supplemented DMEM (to obtain a DNA/PEI ratio of 1:2) followed by 5 min incubation at RT. Next, 0.33 µg hGLP-1R, 0.042 µg Rluc8-Arr3-Sp2, and 0.8 µg mem-citrine-SH3 were combined with the PEI/DMEM mixture followed by 15 min incubation at RT, before the transfection mix was added dropwise to the cells. The transfection was terminated after 24 h by replacing the transfection medium with supplemented DMEM. For the internalization, HEK293A cells (15,000 cells/well) were seeded in a white 384-well plate and transiently transfected with SNAP-hGLP-1R (2.5 ng/well) using Lipofectamine 2000 + Opti-MEM in supplemented DMEM, followed by incubated at 37 °C.

#### cAMP accumulation

One day after the transfection, the transfected cells were seeded in a white 96-well cell culture plate at a density of 35,000 cells/well and incubated for 24 h before the assay. On the assay day, cells were washed with 1xHBS and incubated in 100 µl IBMX (1mM) diluted in 1xHBS for 30 min at 37 °C. Next, 5 µl of GLP-1(7–36)NH_2_ or GLP-1(7–37) was added in concentrations ranging from (1 pM to 10 nM) and incubated for 30 min at 37 °C. Afterward, the assay medium was aspirated, and 30 µL PBS, 10 µl cAMP antibody, and 40 µl enzyme donor/lysis solution were added to each well and incubated for 1 h at RT before 40 µl enzyme acceptor solution was added. After 3 h of incubation at RT, the luminescent signal was measured using a PerkinElmer EnVision 2104 Multilabel Reader.

#### Radioligand competition binding

One day after the transfection, the cells were seeded in a transparent 96-well cell culture plate at a density of 7,500 cells/well and incubated for 24 h before the assay. The number of cells per well was chosen to aim for 5–10% specific binding of the radioligand according to the Cheng-Prusoff Eq. [38]. On the assay day, the cells were washed two times with binding buffer (50 mM HEPES buffer (pH 7.2), 1 mM CaCl_2_, 5 mM MgCl_2_, 0.5% (w/v) BSA) and incubated with binding buffer for 15 min at 4 °C. Unlabeled GLP-1(7–36)NH_2_ or GLP-1(7–37) was added in concentrations ranging from (100 pM to 1 µM), and subsequently [^125^I]GLP-1(7–36)NH_2_ (10.6 pM) was added, followed by 3 h of incubation at 4 °C. Afterward, cells were washed two times with binding buffer and lysed with lysis buffer (200 mM NaOH + 1% SDS). The gamma radiation intensity was measured using a PerkinElmer 2470 WIZARD [2] Automatic Gamma Counter.

#### β-arrestin 2 recruitment

On the assay day, 24 h post transfection, cells were washed with PBS and resuspended in 2 ml PBS + 1% glucose (0.5 M). Subsequently, cells (85 µl/well) were aliquoted into a white 96-well cell culture plate before 10 µl coelentrazine h (5 µM) was added to each well and incubated for 10 min. Next, GLP-1(7–36)NH_2_ or GLP-1(7–37) was added in concentrations ranging from (100 pM to 1 µM). After 30 min incubation at RT, the luminescent signal (535 nm acceptor and 480 nm donor) was measured using a PerkinElmer EnVision 2104 Multilabel Reader.

#### GLP-1R Internalization

24 h post transfection, the transfection medium was replaced with supplemented DMEM, and cells were incubated for 24 h at 37 °C. On the assay day, cells were labeled with the donor 10 µl of SNAP-Lumi4-Tb (0.1 pmol/µl) in Opti-MEM at 37 °C for 60 min. After labeling, the cells were washed four times with internalization buffer (HBSS supplemented with 20 mM HEPES, 1 mM CaCl_2_, 1 mM MgCl_2_). Subsequently, the acceptor, 10 µl of fluorescein-O′-acetic acid (50 µM) in 37 °C internalization buffer, was added to each well, except for the wells used to measure the donor signal. Next, 10 µl of GLP-1(7–36)NH_2_ or GLP-1(7–37) was added in concentrations ranging from (100 pM to 1 µM). Donor and acceptor signals were measured every 3 min at 37 °C using a PerkinElmer Multimode Plate Reader Envision 2105.

## Results

To determine whether PAM loss influences glucose tolerance through defects in the incretin axis we characterized both human and murine in vivo models. First, we confirmed that carriers of the *PAM* loss of function (LoF) alleles p.D563G (MAF ~ 5%, presumed partial LoF) and p.S539W (MAF 1%, presumed complete LoF) have reduced in vivo PAM function by measuring PAM amidation activity in serum from white European individuals without diabetes from the OBB [19]. PAM activity was measured in 24 heterozygous carriers of the p.S539W allele, 27 heterozygous and 21 homozygous carriers of the p.D563G allele and age, sex and BMI matched non-carriers. We observed a 52% reduction in amidation activity in heterozygous carriers of the p.S539W allele compared to non-carriers (188 ± 13 vs. 392 ± 13 pmol/ml/hr *p* = 9.3 × 10^− 15^), (Fig. 1a). Similarly, we observed a 20% (300 ± 11 vs. 370 ± 14 pmol/ml/hr, *p* = 0.0008) and 38% (272 ± 10 vs. 472 ± 17 pmol/ml/hr, *p* = 1.4 × 10^− 9^) decrease in PAM serum activity in heterozygous and homozygous carriers of p.D563G compared to non-carriers, respectively (Fig. 1b).

Concomitantly, to understand how loss of PAM affects systemic processes influencing diabetes onset, we created an inducible *Pam* whole-body knockout mouse model (UBC-Ert2-Cre *Pam*^*fl/fl*^, Additional file 2: Fig. S1a). *Pam*^*fl/fl*^ mice were crossed with mice expressing a tamoxifen-inducible Cre-Ert2 fusion gene under the control of the human ubiquitin C (UBC) promoter [39], and *UBC-Ert2-Cre Pam*^*fl/fl*^ and littermates of genotype *Pam*^*fl/fl*^ were treated with tamoxifen at 4–5 weeks of age to generate *Pam* whole-body knockouts (hereby referred to as PamKO) or control tamoxifen-treated *Pam*^*fl/fl*^ wild type littermates (referred to as WT). Effective *Pam*^*fl/fl*^ allele recombination and lack of Pam expression in PamKO mice was assessed by gene expression and *Pam* ablation in different tissues (Additional file 2: Fig. S1b–d). Supporting the observations in individuals carrying loss of function alleles (Fig. 1a, b), PAM amidation activity was absent in PamKO mice (Fig. 1c, Additional file 2: Fig. S1e). PamKO mice remained normoglycemic but had reduced body weight compared to WT littermate controls (Additional file 2: Fig. S1f–g).

Having demonstrated the functional impact of p.D563G and p.S539W on PAM amidation activity in vivo we assessed the impact of this on postprandial amidated, unamidated and total (sum of amidated and unamidated) plasma GLP-1 concentration in humans. We retrospectively examined amidated GLP-1 levels in two Danish Cohorts (AdditionPRO and Family studies – Additional file 1: Table S1) [22, 40]. In the Family Study, amidated plasma GLP-1 levels (7–36 amide and 9–36 amide) were measured at 10 timepoints following an OGTT. We compared GLP-1 profiles and the AUC_120_ in 26 Danish carriers of the p.D563G allele and 56 matched non-carriers (Fig. 1d) [19, 20]. The peak amidated GLP-1 concentration (highest value at any time point) was higher in the carriers of the p.D563G allele (17.7 ± 1.3 vs. 23.9 ± 3.4 pmol/L, *p* = 0.04), as was the overall postprandial exposure, measured by GLP-1 AUC_120_ (2498 ± 168 vs. 3251 ± 303 pmol.L^− 1^.min, *p* = 0.02). Only 3 heterozygous carriers of the p.S539W allele were identified in the Family study participants. This study was underpowered to detect a difference in carriers of p.S539W and did not demonstrate a significant difference (1340 ± 220 vs. 1895 ± 62 pmol.L^− 1^.min., *p* = 0.13) (Fig. 1e). Additionally, we prospectively measured GLP-1 7–36 amide and GLP-1 7–37 Gly in stored plasma from the Oxford Biobank, this demonstrated no difference in fasting GLP-1 7–36 amide and GLP-1 7–37 Gly concentration or the ratio between them in heterozygous carriers of p.S539W and non-carriers (Additional file 2: Fig. S2a).

The observation of increased post-prandial GLP-1 concentration and increased risk of T2D in carriers of *PAM* diabetes risk alleles is counterintuitive with prior studies which demonstrated reduced GLP-1 concentrations in individuals with pre-diabetes and diabetes compared with persons without diabetes [41, 42], although this pattern has been reported in carriers of *TCF7L2* diabetes risk alleles who have been termed “incretin resistant”^43^. We sought to prospectively re-confirm the impact of PAM deficiency on postprandial GLP-1 levels and secondly assess the consequence of altered GLP-1 levels on the incretin response using an isoglycemic clamp, the gold standard measure of the incretin response. We performed a recruit-by-genotype study in 19 white European normoglycemic carriers of the presumed complete LoF allele p.S539W from the OBB and on 19 age, sex and BMI matched non-carriers. We measured amidated and glycine extended GLP-1 concentration (both of which are biologically active) (Additional file 2: Fig. S2b-e) and calculated the total GLP-1 concentration at 10 time points following a 75 g oral glucose load. The total (sum of amidated and unamidated) GLP-1 profiles were significantly higher in carriers compared to non-carriers (p = 0.035) (Fig. 1f). The mean AUC_240_ was higher in carriers compared to non-carriers (Total GLP-1: 7692 ± 304 vs. 6887 ± 230 min.pmol/L p = 0.04). Importantly, there were no significant differences in insulin or glucose profiles between genotypes or ratio of amidated to non-amidated GLP-1 at any time point (Table 1).

To establish whether differences in binding affinity could contribute to the elevated GLP-1 levels in *PAM* T2D-risk allele carriers we measured receptor binding affinity in competition with ^125^I-GLP-1(7–36 amide) for both the amidated and non-amidated forms of GLP-1 demonstrating that they are equipotent (Additional file 2: Fig. S2b). An assessment of agonist signaling bias also showed no differences in cAMP accumulation, receptor internalization or beta arrestin recruitment between amidated and non-amidated GLP-1 (Additional file 2: Fig. S2c-e).

Similar to our observations in human carriers of *PAM* LoF alleles, PamKO mice exhibited higher total GLP-1 levels in the fasted state and after 10 min of an oral glucose load (Fig. 1g, h). To assess whether this elevation was attributable to altered GLP-1 secretion we also measured GLP-2, which is encoded carboxyterminal to the GLP-1 sequence in the proglucagon gene and co-secreted with GLP-1 from intestinal L-cells [26], and found that it was also increased in the blood of PamKO mice (Fig. 1i). The expression and content of GLP-1 in the duodenum, jejunum and colon of PamKO mice was similar to control littermates (Additional file 2: Fig. S3a–e), and GLP-1 immune staining revealed similar L-cell density in villi of the ileum and colon (Additional file 2: Fig. S3f, g). Furthermore, plasma DPP4 activity was unchanged between the two groups (Additional file 2: Fig. S3h). These data indicate that the increase in GLP-1 after glucose challenge or feeding in mice lacking *Pam* is not due to altered L-cell densities, differences in GLP-1 production or DPP4 activity, but rather to altered GLP-1 secretion.

**Fig. 2:**
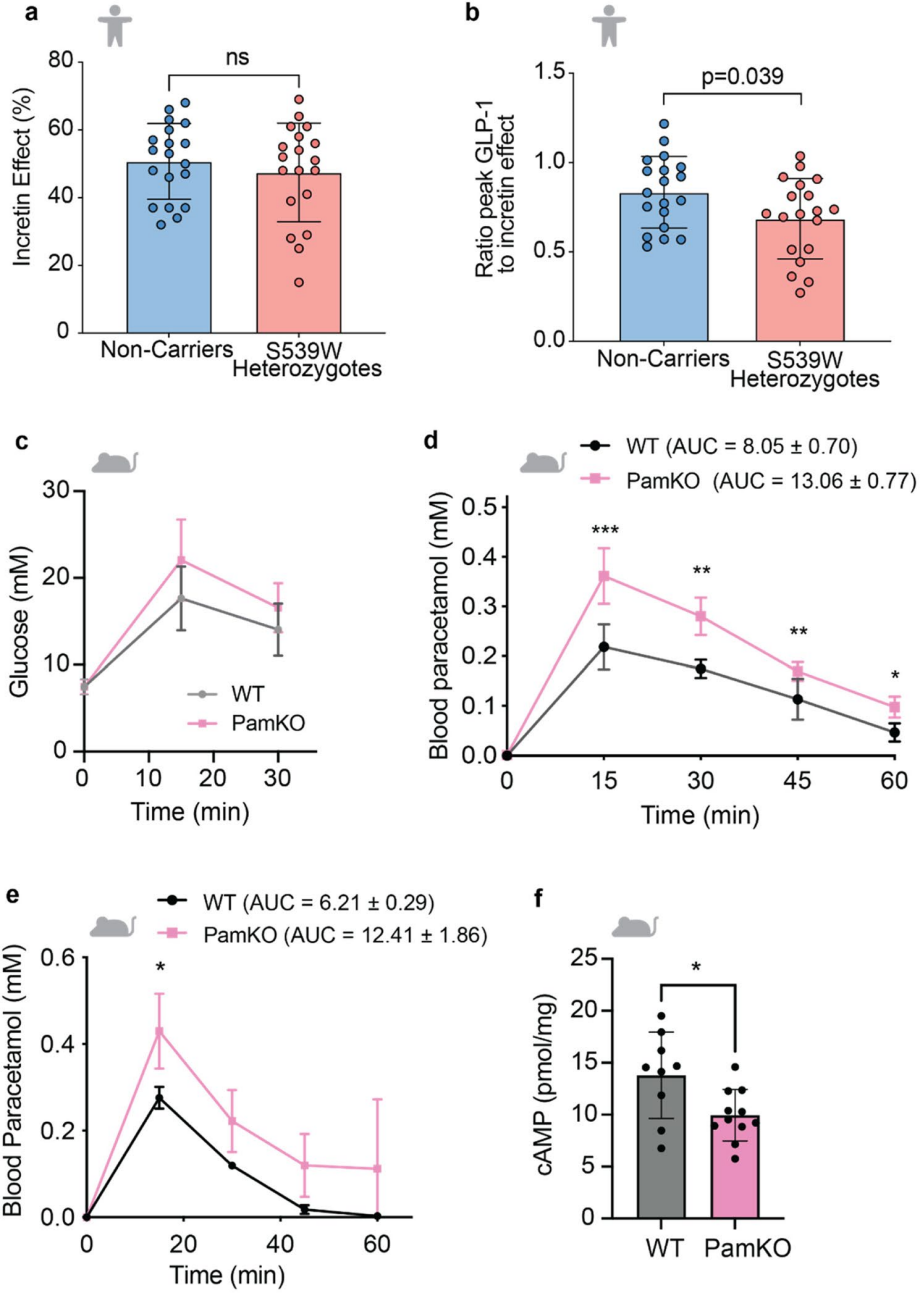
PAM loss leads to GLP-1 Resistance. **a** Incretin effect in 19 heterozygous carriers of p.S539W and 19 matched non-carriers as measured by the IV glucose required as a percentage of 75 g to re-produce a glucose curve associated with a 75 g oral glucose load. Error bars represent SD. **b** Ratio between peak GLP-1 concentration and incretin effect in 19 heterozygous carriers of p.S539W and 19 matched non-carriers. This is a surrogate of GLP-1 sensitivity and demonstrate a significant reduction in GLP-1 sensitivity in carriers of p.S539W compared to non-carriers. Error bars represent SD. **c** Glucose tolerance during oGTT in PamKO and WT littermates (*n* = 7,8). **d** Plasma paracetamol levels at indicated timepoints after an oral paracetamol load as a surrogate for gastric emptying rate in PamKO and WT littermates (*n* = 7,7). Area under the curve (AUC) is indicated ± standard error. **e** Gastric emptying rate during treatment with GLP-1 receptor agonist (exendin-4) as measured by the paracetamol absorption test (*n* = 4,8). Area under the curve (AUC) is indicated ± standard error. **f** cAMP levels in pylorus of PamKO and WT littermate mice, measured 3 min after exenatide 4 injection (*n* = 11,9). Data are presented as mean ± SD; two tailed *t* test (**f**) and 2-way repeated measures ANOVA with Sidak’s multiple comparisons test (**c**–**e**), *P < 0.05, **P < 0.01, ***P < 0.001

Having demonstrated elevated GLP-1 concentrations in carriers of PAM LoF alleles and *Pam* Knockout mice, we assessed the functional impact on postprandial glucose homeostasis by measuring the incretin effect. The incretin effect refers to the higher insulin secretion observed when glucose is delivered orally vs. intravenously when glycemia is matched and has been attributed to the action of gut released peptides, GLP-1 and glucose dependent insulinotropic polypeptide (GIP) [20]. We quantified the incretin effect with the gold standard isoglycemic clamp in the same 19 p.S539W carriers and 19 matched non-carriers from the OBB [20]. Despite higher postprandial GLP-1 concentrations there was no difference in the incretin response in carriers compared to non-carriers (47.5% ± 14.4 vs. 50.7% ± 11.3, *p* = 0.50) (Fig. 2a). To quantify GLP-1 action, we compared the ratio of incretin effect to the peak total (amidated and non-amidated) GLP-1. We found a 18% reduction in GLP-1 sensitivity as measured by GLP_peak_:incretin effect ratio in carriers of p.S539W compared to non-carriers (0.69 ± 0.05 vs. 0.83 ± 0.04, *p* = 0.04) (Fig. 2b). To exclude a contribution of GIP (the major contributor to the incretin effect in health) to the observed phenotype, GIP AUC was also assessed after measurement at 10 time points, no difference was observed between p.S539W allele carriers and non-carriers (11670 ± 5518 vs. 11781 ± 4725 min.pmol/L, *p* = 0.95) (Table 1). There were also no differences at baseline or at maximal stimulation in gastrin-Gly, gastrin amide, CCK-amide, Islet Amyloid PolyPeptide (IAPP)-Gly and IAPP-amide levels between carriers and non-carriers (Extended Data Fig. 4; Table 1). The consistent observation of elevated circulating GLP-1 levels in the absence of a biological effect is a hallmark of GLP-1 resistance and analogous the “incretin resistance” reported in carriers of *TCF7L2* T2D-risk alleles [43]. The absence of evidence for direct feedback from the beta cell on GLP-1 concentration suggests that the “resistance” is at least in part external to the beta cell.

To further delineate the mechanism of PAM deficiency on GLP-1 resistance, pancreas-specific (Pdx-Cre *Pam*^*fl/fl*^) and induced whole body knockout mice (PamKO) were studied. Both models exhibited similar postprandial glycemia during an OGTT, with similar plasma insulin levels compared to littermate controls, suggesting impaired GLP-1 action on the endocrine pancreas (Fig. 2c, Additional file 2: Fig. S5a, b). Gastric emptying rate (GE), the predominant GLP-1 mechanism mediating postprandial glycemic control, was measured by a paracetamol absorption test in PamKO mice [44]. Ablation of *Pam* caused an accelerated GE rate in PamKO mice compared to control animals (Fig. 2d, AUC WT 8.05 ± 0.70 vs. AUC PamKO 13.06 ± 0.77 mM.min) which when treated with Exendin-4, at a dose previously demonstrated to robustly slow GE (10 nmol/kg) revealed that the GE rate of PamKO mice remained faster than wildtype controls during GLP-1 treatment [45] (Fig. 2e, AUC WT 6.21 ± 0.29 vs. AUC PamKO 12.41 ± 1.86 mM.min). Given several lines of evidence for GLP-1 resistance (elevated GLP-1 levels in the absence of a biological effect on glycemia or GE), we analyzed transcript expression of the GLP-1 receptor (*Glp1r*) and monitored GLP-1 receptor signaling by measuring cAMP levels following administration of Exendin-4 in the gastric pylorus, which influences GE rate. While the expression of *Glp1r* in the pylorus was similar, levels of cAMP in response to GLP-1 stimulation were decreased in the pylorus of PamKO mice compared to wildtype littermates (Fig. 2f, ). Furthermore, reduction in *Glp1r* expression was not observed in the other tissues tested (Additional file 2: Fig. S5d-h), whilst expression of other gastric hormone receptors in the pylorus, except for GRP were unchanged, (Additional file 2: Fig. S5i-j). Together, these data suggest that impaired GLP-1 post-receptor signaling in the pylorus contributes to the increased GE rate, potentially via reduced effect on pyloric sphincter tone in mice lacking *Pam*.

**Fig. 3:**
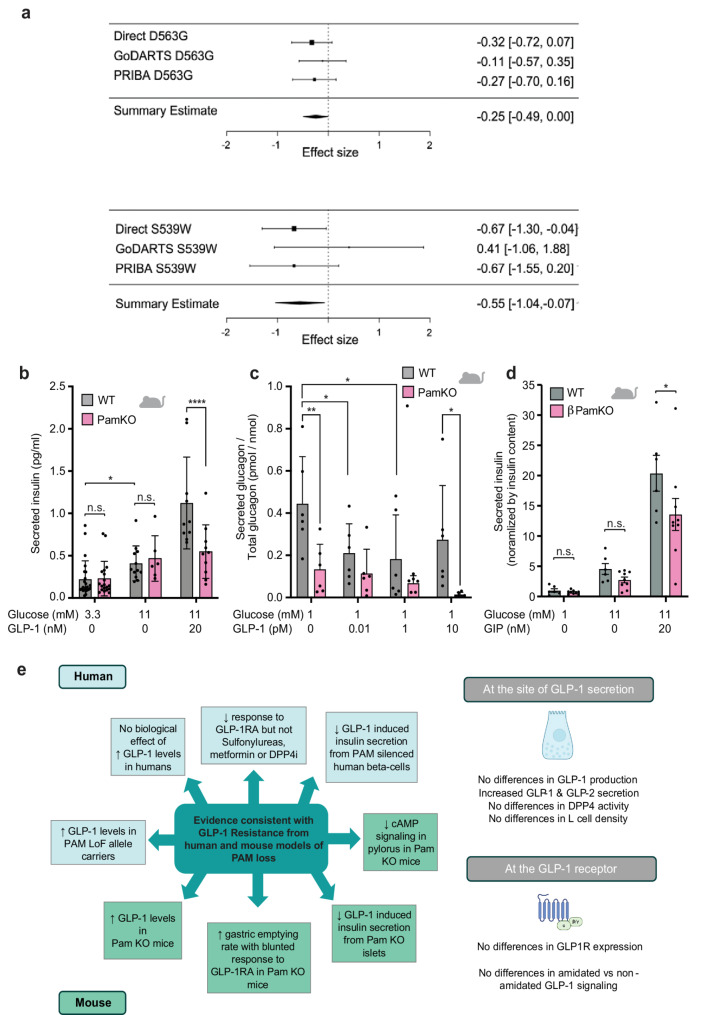
Meta-analysis of the effect of carrying LoF alleles at p.D563G and p.S539W on response to GLP-1RA therapy and decreased pancreatic islet cell responses in response to exenatide 4 exposure. **a** The effect of carrying p.D563G and p.S539W on treatment response to GLP-1RA. Each study is displayed separately, and the effect size is indicated by the location of a solid box with the 95% CI displayed either side. The line of no effect is indicated by a vertical dotted line. The summary estimate of the effect of each allele is displayed below the individual cohort summaries and is indicated by a solid black diamond with the center of the diamond indicating the summary estimate and the lateral points the 95% CI. The effect displayed is the mean absolute HbA1c change between baseline and 6 months (% DCCT units). The meta-analysis comprises 1,119 individuals across the 3 studies 130 carrier p.D563G and 26 carriers of pS539W. **b**, Insulin secretion of isolated islets from PamKO and WT littermate mice that were stimulated for 1 h at low (3.3 mM) or high (11 mM) glucose or high glucose and GLP-1 (20 nM) (*n* = 24,12,12 for WT, and *n* = 20,8,12 for PamKO, respectively). **c**, Glucagon secretion of isolated islets from PamKO and WT littermate mice that were cultured for 1 h at low (1 mM) glucose and in the absence or increasing concentrations of GLP-1 (*n* = 6,6). **d** Insulin secretion of isolated islets from beta cell specific PamKO (βPamKO, *n* = 9) and WT littermate mice (*n* = 6) that were stimulated for 1 h at low (1 mM) or high (11 mM) glucose or high glucose and GIP (20 nM). Data are presented as mean ± SD; 2-way repeated measures ANOVA with Sidak’s multiple comparisons test (b, c), *P < 0.05, **P < 0.01, ****P < 0.001; n.s. *p* > 0.05. **e** Summary of mouse and human evidence that support GLP-1 resistance upon loss of PAM and impaired hormone amidation (left) and overview for the role of PAM and peptide amidation at the GLP-1 receptor and at the site of GLP-1 secretion (right)

Evidence of GLP-1 resistance in the physiological setting prompted examination of the therapeutic response to GLP-1RA. A meta-analysis of 1,119 study participants treated with GLP-1RAs in 3 investigator led cohorts (Innovative Medicines Initiative – DIabetes REsearCH on patient straTification (IMI-DIRECT), Genetics of Diabetes Audit and Research in Tayside Scotland (GoDARTS) and Predicting Response to Incretin Based Agents [PRIBA]) was performed, where HbA1c was measured at initiation of GLP-1RA treatment and at 6 months. Individuals in these studies were treated with liraglutide, twice-daily exenatide or once-weekly exenatide. In non-carriers of the *PAM* LoF alleles the mean absolute HbA1c change across the 3 studies after GLP-1 treatment was − 1.24% (13.6 mmol/mol). The meta-analysis demonstrated a significant reduction in HbA1c with 6 months of therapy with GLP-1RA in both carriers and non-carriers of *PAM* LoF alleles (Fig. 3a). The absolute magnitude of this reduction was significantly less in heterozygous carriers of the p.S539W *PAM* allele with a mean change in HbA1c of -0.69% (-7.5 mmol/mol). This amounted to an absolute loss of 0.55% (6.0 mmol/mol) HbA1c lowering per allele, *p* = 0.025. A suggestive albeit non-significant difference in absolute HbA1c lowering was observed in heterozygous carriers of p.D563G after GLP-1RA initiation (-0.25% (2.7 mmol/mol) per allele, *p* = 0.050). The meta-analysis demonstrated a clinically meaningful reduction in this response to GLP-1RA in both carriers of the p.S539W or the p.D563G (Fig. 3a). This represents a relative loss of either 44% or 20% of the HbA1c lowering associated with GLP-1 RA use respectively. Of note, in the DIRECT study, carriers of p.S539W had an independently significant reduction in response to GLP-1RA (-0.67% (7.3 mmol/mol) per allele, *p* = 0.034). In these studies, 11.5*%* of p.S539W carriers and 18.5% of p.D563G carriers in whom GLP-1RA treatment was initiated achieved the recommended HbA1c target of < 7% compared to 25.3% of non-carriers.

To determine if a reduced response to GLP-1RA was driven by a GLP-1 specific effect we also determined the effect of *PAM* genotype on the response to three additional commonly used anti-diabetic drugs, DPP-4i, metformin, and sulphonylureas, in the same studies. No significant differences were detected in response to any these medications between carriers and non-carriers of the *PAM* LoF alleles (Additional file 1: Table S3-5 and Additional file 2: Fig. S6).

We then sought to reproduce this finding by examining the effect of *PAM* genotype on response to once-weekly albiglutide therapy (30-50 mg) in the GSK-Harmony trial and once-weekly exenatide therapy (2 mg) in the EXSCEL study [32], [33]. The GSK-Harmony data were not included in the meta-analysis due to substantial methodological differences (the option to intensify GLP-1RA therapy in non-responders, measurement of effect over 8 months rather than 6 months and the relatively small glycemic effect seen with albiglutide). Both carriers and non-carriers of *PAM* LoF alleles again demonstrated a reduction in HbA1c at 8 months. The magnitude of the reduction was 0.63%, approximately half that seen in the meta-analysis. There was no difference in response to GLP-1RA between carriers and non-carriers of the *PAM* p.S539W (0.28% (3.1 mmol/mol), SE:0.18, *p* = 0.12) and p.D563G (-0.04% (0.4 mmol/mol), SE:0.07, *p* = 0.53) alleles. The EXSCEL cardiovascular outcome trial data were also not included in the meta-analysis due to similar methodological differences. This analysis had similar limitations with not all the variables included in the meta-analysis being available and direct genotyping only available for the p.D563G allele, imputation of p.S539W was available, but is not reliable. The additive multiple linear regression model, including baseline HbA1c level as a covariate, did not demonstrate a significant effect of genotype on glycemic control at 6 months (0.037%, 95%CI [-0.213, 0.287], *p* = 0.77).

Given the inconsistent effect observed in the GSK-Harmony and EXSCEL trials and the meta-analysis, which comprised of 3 different GLP-1RAs, we sought to assess agonist-specific effects. Retrospective analysis by agonist, although underpowered, and comprising different study designs demonstrated a reduced response to liraglutide in carriers of p.D563G but not p.S539W (p.D563G − 0.25% (2.7 mmol/mol), *p* = 0.05 & pS539W -0.16%, *p* = 0.55). Response to twice-daily exenatide was reduced in carriers of p.S539W but not p.D563G (p.D563G − 0.19% (2.0 mmol/mol), *p* = 0.41 & pS539W -0.84% (9.0 mmol/mol), *p* = 0.035). There was no difference in response to albiglutide in either of the *PAM* T2D-risk allele carriers (p.D563G − 0.03% (0.3 mmol/mol), *p* = 0.73 & pS539W 0.30% (3.3 mmol/mol), *p* = 0.15). (Additional file 2: Fig. S7).

We also assessed potential differences in response to long-acting compared to short-acting GLP-1RAs, given reported difference on gastric emptying between short and long-acting agonist [46]. Agonists that resulted in continuous GLP-1 receptor activation were categorized as long-acting and those that had periods without receptor activation were categorized as short-acting. Only twice-daily exenatide was categorized as short-acting. Whilst this exploratory analysis was underpowered, there was a significant reduction in response to twice-daily exenatide in carriers of p.S539W compared with non-carriers (-0.84%, 95%CI [-1.56, -0.12], *p* = 0.023), whilst directionally consistent there was no significant difference between carriers of p.D563G (-0.19%, 95% CI [-0.66, 0.27], *p* = 0.41). When response to long-acting GLP-1RA were compared, there was no difference between carriers of p.S539W and control (0.05%, 95% CI [-0.26, 0.36], *p* = 0.46) or p.D563G and control (-0.07%, 95%CI [-0.18, 0.04], *p* = 0.22).

Given the reduced efficacy of GLP-1RAs on glycaemia and the reported association of genetic variation in *PAM* with BMI we sought to establish whether the reduced efficacy extended to effects on weight loss in the two studies (PRIBA and GSK-Harmony) where data were available following 6 months of treatment (Additional file 2: Fig. S8). In the modest dataset available there were no significant differences in weight loss for either p.S539W (-0.58 kg, 95%CI [--2.01,0.85]) or p.D563G (-0.27 kg, 95%CI [-0.82,0.29]) in the meta-analysis of the GSK Harmony trial and the PRIBA Study.

To further explore the pancreatic response to GLP-1, we assessed the response to GLP-1RA treatment in isolated islets from PamKO mice and wildtype littermates. Whilst isolated mouse islets from both genotypes had similar responses when challenged with high glucose, PamKO islets had a blunted response when stimulated with high glucose and GLP-1 (Fig. 3b). To assess the specificity of the blunted secretory response of the islet to GLP-1, response to GIP (which would suggest or a general post receptor signaling defect) was compared in wildtype and PamKO mice. PamKO islets also had a blunted response when stimulated with high glucose and GIP consistent with a generalized post-receptor defect (Fig. 3d). To explore if PAM loss also influences the GLP-1 mediated inhibitory effect of glucagon secretion, we cultured isolated pancreatic islets of PamKO and control mice in low glucose and increasing doses of GLP-1. Glucagon secretion from PamKO islets was reduced at low glucose levels in the absence of GLP-1 and importantly did not significantly suppress secretion at increasing GLP-1 levels compared to littermate control mice (Fig. 3c). As GLP-1 has not been shown to suppress glucagon in hypoglycemia in stepped hypoglycemia clamps [47], an observation contrary to prior pancreatic perfusion studies [48], we also assessed glucagon concentration in carriers and non-carriers of p.S539W alleles during OGTT. Consistent with our data in PamKO islets, in humans, during an OGTT there was a strong interaction between genotype and time on glucagon concentration (*p* = 0.0009). Post hoc pairwise testing demonstrated a significant difference (without adjustment for multiple testing) at 0, 15, 30 min of the OGTT with an average increase in the p.S539W group of 3.1 pmol/L during this time (Additional file 2: Fig. S9a). We also determined whether a diminished response to GLP-1RA could be observed in *PAM* silenced human beta cells using siRNA mediated knockdown in EndoC-βh1 cells. Despite seeing no difference between baseline insulin and glucose secretory responses we did observe a reduced response to GLP-1 potentiated insulin secretion in cells with reduced *PAM* expression consistent with our murine model (Additional file 2: Fig. S9b). These data indicate in addition to effects on GE, PAM loss also influences the responsiveness of pancreatic beta and alpha cells to GLP-1.

## Discussion

Translating genome wide association signals in T2D, into clinically useful information has been challenging. In this study we demonstrate how in-depth physiological characterization of a GWAS signal can lead to biological insight and subsequent focused examination of pharmacogenetic studies can provide implications for a currently available treatment. We demonstrated in multiple human studies that two LoF alleles in *PAM* resulted in reduced PAM activity and increased post-prandial GLP-1 levels in the absence of an improved incretin response and leading to a reduced response to GLP-1RA (Fig. 3e). These observations are consistent with PAM LoF allele carriers exhibiting GLP-1 resistance.

Further support for GLP-1 resistance was obtained in mouse models with genetic *Pam* inactivation (Fig. 3e). Mice lacking *Pam* exhibited accelerated GE and a blunted response to GLP-1-mediated slowing of GE compared to control mice. Following GLP1RA binding, GLP1R engages Gαs to activate adenylate cyclase (AC), thus generating cAMP [49, 50]. Pyloric cAMP production was reduced in PamKO mice after GLP-1 stimulation, indicating an altered PAM associated post-GLP1R signaling effect. This may also suggest a mechanism by which the gastric slowing is attenuated in carriers of hypomorphic *PAM* alleles. Muscle contraction in the pyloric sphincter resists the pressure placed on gastric content by antral contraction [51]. Reduced cAMP in the pylorus could indicate reduced muscle contractility and may explain the more rapid GE observed in mice in the physiological setting and during pharmacological exposure to exendin-4. As pyloric muscle tone and GE rate is under control of both central nervous system and peripheral regulation, the observation of reduced pyloric cAMP could relate to GLP-1 resistance centrally or peripherally.

Evidence for GLP-1 resistance was also obtained in pancreatic islets, where in PamKO we observed reduced stimulation of insulin secretion and reduced suppression of glucagon release in response to GLP-1 exposure. We also observed a similar blunting of insulin secretion during GIP and high glucose exposure. Both GIP and GLP-1 share a similar post receptor signaling pathway to stimulate insulin secretion. This supports PAM playing a role in the post receptor signaling in GLP-1/GIP mediated insulin secretion. Notably, in these models, PamKO resulted in no difference in insulin secretion at high glucose without incretin exposure. This suggests that PAM may be interacting with an insulin secretory pathway that is specific to the incretin pathway. Potential sites of interaction include dampening of cAMP or granule accumulation. This is consistent with our cohort level observation of reduced GLP-1R effect in *PAM* LoF carriers but no difference of effect of sulphonylureas between genotypes and our previous work identifying altered levels of amidated peptides (Chromogranin A) involved in intracellular trafficking and the observation of altered kinetics of exocytosis [12]. We acknowledge however, that we have not excluded an effect of PAM dysfunction on GLP-1 clearance nor have we fully determined the precise contributions of PAM loss from different tissues on glucose homeostasis. The exclusive role of PAM in the amidation of peptides which are central to homeostatic regulation creates a complex system to dissect as evidenced by the well reported associations of *PAM* variants in humans with BMI and waist-hip-ratio and the reduction in body weight and normoglycemia observed in our global inducible Pam KO mouse [18].

The precise physiological and molecular consequences of how a loss of PAM affects GLP-1 levels and signaling is likely to be complex since so many hypothalamic and peripherally secreted peptides involved in the regulation of energy homeostasis are amidated [52] and a central action of PAM in GLP-1 resistance can be ruled out [53]. Furthermore, as Cu^2+^, Zn^2+^ and ascorbate are essential cofactors of PAM activity [53], levels of these factors could further impact the effect of partial PAM deficiency and GLP-1 resistance in humans. Therefore, it will be interesting in future studies to explore if different blood levels of these cofactors can be linked to the severity of GLP-1 resistance in individuals with hypomorphic T2D-risk alleles [53]. Future studies investigating the effects of PAM LoF on food intake and body weight will be required to elucidate the extent to which effects of elevated circulating GLP-1 levels extend beyond glycemic control and to provide greater mechanistic insight.

We observed differing results between the examination of cardiovascular outcome trials using the long-acting GLP-1RAs, (once-weekly exenatide and albiglutide) and shorter-acting GLP-1RAs in the investigator led trials (twice-daily exenatide). Whilst this may reflect substantial methodological differences evidenced by a signal for reduced efficacy of liraglutide in the investigator led trials, it may also be reflective of the difference between the degree of gastric slowing observed with short and long-acting GLP-1RAs. This is particularly intriguing given that our murine data support effects of Pam loss on GE. It has been demonstrated that continuous GLP-1R stimulation results in rapid tachyphylaxis to the gastric slowing effects of GLP-1 but not the pancreatic effects, however with intermittent stimulation of the GLP-1R with shorter-acting agonist gastric slowing is maintained [54]. As such the impact of attenuating the gastric slowing may be more marked in shorter-acting agonists and may explain why no effect was observed in with albiglutide or once-weekly exenatide.

The glycemic impact of DPP-4 inhibitors was unaffected by *PAM* genotype. This may reflect the absence of effect of DPP-4 on GE or the higher circulating concentrations of GLP-1 observed in *PAM* carriers may augment the effect of DPP-4i in prolonging the half-life of GLP-1, which may off set the expected GLP-1 resistance [55].

Given that therapeutic inertia has been shown to increase rates of diabetic complications, this raises concern about the use of GLP-1 receptor agonists in carriers *PAM* LoF alleles [4]. Importantly, we demonstrate that there was no impact of *PAM* genotype on response to metformin, sulphonylurea, or DPP-4i alternate medication choices in individuals without heart disease or renal impairment. As pharmacogenetic cohorts increase in size and more alleles are identified which predict treatment response, a likely development will be the development of polygenic risk scores which predict likely response of an individual to the various diabetes agents.

## Conclusions

In conclusion, examination of the T2D *PAM* locus revealed that carriers of LoF alleles have reduced serum enzyme activity, and elevated circulating levels of GLP-1 in the absence of a biological effect. The “GLP-1 resistance” in these carriers resulted in a specific and clinically meaningful reduction in response to exenatide and liraglutide. Data from a *Pam* knockout mouse suggests that the mechanism of GLP-1 resistance is linked to accelerated GE and resistance to GLP-1 in the endocrine pancreas.

## Supplementary Information

Supplementary Material 1: contains supplementary methods, supplementary Tables 1–5, pharmacogenetic cohort details and study plan.

Supplementary Material 2: contains supplementary Figs. 1–9.

## Data Availability

Data generated or analysed during this study are included in this published article (and its supplementary information files). IMI-DIRECT data access is available on request (DIRECTdataaccess@dundee.ac.uk). HARMONY data can be requested via clinicalstudydatarequest.com. De-identified participant-level data generated in this study from the Oxford Biobank can be requested from the corresponding author pending approval from the Oxford Biobank. Requests for access to EXSCEL trial data should be submitted viahttps://vivli.org/members/enquiries-about-studies-not-listed-on-thevivli-platform/.
