# Supplementary material for: Type 2 diabetes risk alleles in peptidyl-glycine alpha-amidating monooxygenase influence GLP-1 levels and response to GLP-1 receptor agonists
Source: Genome Med. 2026 Mar 29;18:40. doi: 10.1186/s13073-026-01630-0 (PMC13072570; doi:10.1186/s13073-026-01630-0)
Supplement: Supplementary file 1 — Supplementary Material 1: contains supplementary methods, supplementary Tables 1–5, pharmacogenetic cohort details and study plan. [file 13073_2026_1630_MOESM1_ESM.docx]

**Section 1: Supplemental methods**

**PAM amidation assay**

A radioisotope based kinetic assay was adapted from Mizuno et al. 0.5 mM Ascorbate and 4.0 μM CuSO4 (two required co-factors for PAM), 0.5 μM Ac-Tyr-Val-Gly (unlabelled precursor), 20,000 DPM I125- Ac-Tyr-Val-Gly (labelled precursor), 150 mM Na MES (pH5.5) (the optimum pH for PAM activity), and 0.1 mg/ml Catalase (to scavenge reactive oxygen species). 50 μL of mastermix was combined with 4μL of serum. This was then incubated at 37 °C for 1 hour. The reaction was then stopped by transferring the samples into an ice bath and adding 5 μL of 0.5 mM EDTA pH 8.0. The total amount of I125 was then determined by counting each sample for 5 minutes on the Wizzard2 Gamma Counter (Perkin Elmer, Waltham, USA) using the raw counts program. We then extracted the amidated fraction by adding 700 µL of fresh water-saturated ethyl-acetate and vortexing for 5 seconds. This dissolved the amidated product of PAM but not the unamidated product. As ethyl acetate containing the amidated product separated into a different phase from the mastermix (the upper phase), 350 µl of the top phase was place this into a new RIA tube. The decay/minutes in the tube were then measured in the gamma counter. The amidation activity was then determined using the equation below.

$\mathrm{PAM} \mathrm{Activity}=\frac{\left( 2\times amidated dpm \right)-(2\times blank dpm)}{20,000 (or average total counts)}\times\frac{5000(substrate in pmol)}{1 (time in hrs)}\times\frac{1}{4 (vol serum uL)}$

**Section 2: Supplementary Tables 1–5**

**Table S1. Retrospective examination of amidated GLP-1 levels**

Family Study Demographics

|  | Non-carrier | p.S539W | P value |
| --- | --- | --- | --- |
| n (m/f) | 48 (20/28) | 24 (10/14) | 1.00 |
| Age, mean (SE) | 38.46 (1.52) | 36.95 (2.38) | 0.58 |
| BMI, mean (SE) | 26.43 (0.58) | 26.53 (0.84) | 0.92 |

|  | Non-carrier | p.S539W | P value |
| --- | --- | --- | --- |
| n (m/f) | 6 (6/0) | 3 (3/0) | 1.00 |
| Age, mean (SE) | 37.43 (1.65) | 38.19 (3.64) | 0.83 |
| BMI, mean (SE) | 22.92 (0.79) | 22.95 (1.24) | 0.99 |

Addition-Pro Study Demographics

|  | Non-carrier | p.S539W | P value |
| --- | --- | --- | --- |
| n (m/f) | 14 (6/8) | 7 (3/4) | 1.00 |
| Age, mean (SE) | 62.7 (1.6) | 62.8 (2.4) | 0.95 |
| BMI, mean (SE) | 24.3 (0.6) | 24.3 (0.9) | 0.96 |

|  | Non-carrier | p.D563G | P value |
| --- | --- | --- | --- |
| n (m/f) | 290 (148/142) | 145 (74/71) | 1.00 |
| Age, mean (SE) | 66.6 | 66.5 | 0.91 |
| BMI, mean (SE) | 27.6 | 27.6 | 1.00 |

**Table S2: Pharmacogenetic Cohort Details**

Participants were excluded from meta-analysis if genotypic information or treatment response information was not available. If HbA1c was available at 3 months but not 6 months this value was used as a surrogate.

| Cohort | Number of participants | MAF (%) | Beta | SE |
| --- | --- | --- | --- | --- |
| DIRECT | 354 | 5.7% | -0.32 | 0.20 |
| GoDARTS | 291 | 7.7% | -0.11 | 0.24 |
| PRIBA | 466 | 4.8% | -0.27 | 0.22 |

| Cohort | Number of participants | MAF (%) | Beta | SE |
| --- | --- | --- | --- | --- |
| DIRECT | 354 | 1.7% | -0.67 | 0.32 |
| GoDARTS | 297 | 0.67% | 0.41 | 0.75 |
| PRIBA | 468 | 1.1% | -0.67 | 0.45 |

**Table S3 Effect of *PAM genotype on* metformin response**

| **SNP** | **MAF** | **β(SE)** | **P** |
| --- | --- | --- | --- |
| rs35658696 | 0.067 | -0.035 (0.056) | 0.52 |
| rs78408340 | 0.015 | 0.16 (0.11) | 0.15 |

n=2463

**Table S4 Effect of *PAM genotype on* sulphonylurea response**

| SNP | **MAF** | **β(SE)** | **P** |
| --- | --- | --- | --- |
| rs35658696 | 0.067 | 0.049 (0.058) | 0.40 |
| rs78408340 | 0.013 | 0.0033 (0.126940) | 0.98 |

n=2282

**Table S5 : Meta-analysis of DPP-IVi by genotype**

| **Cohort** | **Number in cohort** | **MAF (%)** | **β(SE)** |
| --- | --- | --- | --- |
| GoDARTS 1% | 511 | 1.4% | -0.09 (0.31) |
| PRIBA 1% | 245 | 1.2% | 0.82(0.50) |
| GoDARTS 5% | 487 | 5.7% | 0.17(0.15) |
| PRIBA 5% | 244 | 5.5% | 0.003 (0.24) |

**Section 3: Pharmocogenetic cohort details**

**GLP-1RA response cohort description**

Participants were excluded from meta-analysis if genotypic information or treatment response information was not available. If HbA1c was available at 3months but not 6 months this value was used as a surrogate.

p.D536G

| Cohort | Number of participants | MAF (%) | Beta | SE |
| --- | --- | --- | --- | --- |
| DIRECT | 354 | 5.7% | -0.32 | 0.20 |
| GoDARTS | 291 | 7.7% | -0.11 | 0.24 |
| PRIBA | 466 | 4.8% | -0.27 | 0.22 |

p.S539W

| Cohort | Number of participants | MAF (%) | Beta | SE |
| --- | --- | --- | --- | --- |
| DIRECT | 354 | 1.7% | -0.67 | 0.32 |
| GoDARTS | 297 | 0.67% | 0.41 | 0.75 |
| PRIBA | 468 | 1.1% | -0.67 | 0.45 |

**Meta-analysis of DPP-IVi by genotype**

| **Cohort** | **Number in cohort** | **MAF (%)** | **β(SE)** |
| --- | --- | --- | --- |
| GoDARTS 1% | 511 | 1.4% | -0.09 (0.31) |
| PRIBA 1% | 245 | 1.2% | 0.82(0.50) |
| GoDARTS 5% | 487 | 5.7% | 0.17(0.15) |
| PRIBA 5% | 244 | 5.5% | 0.003 (0.24) |


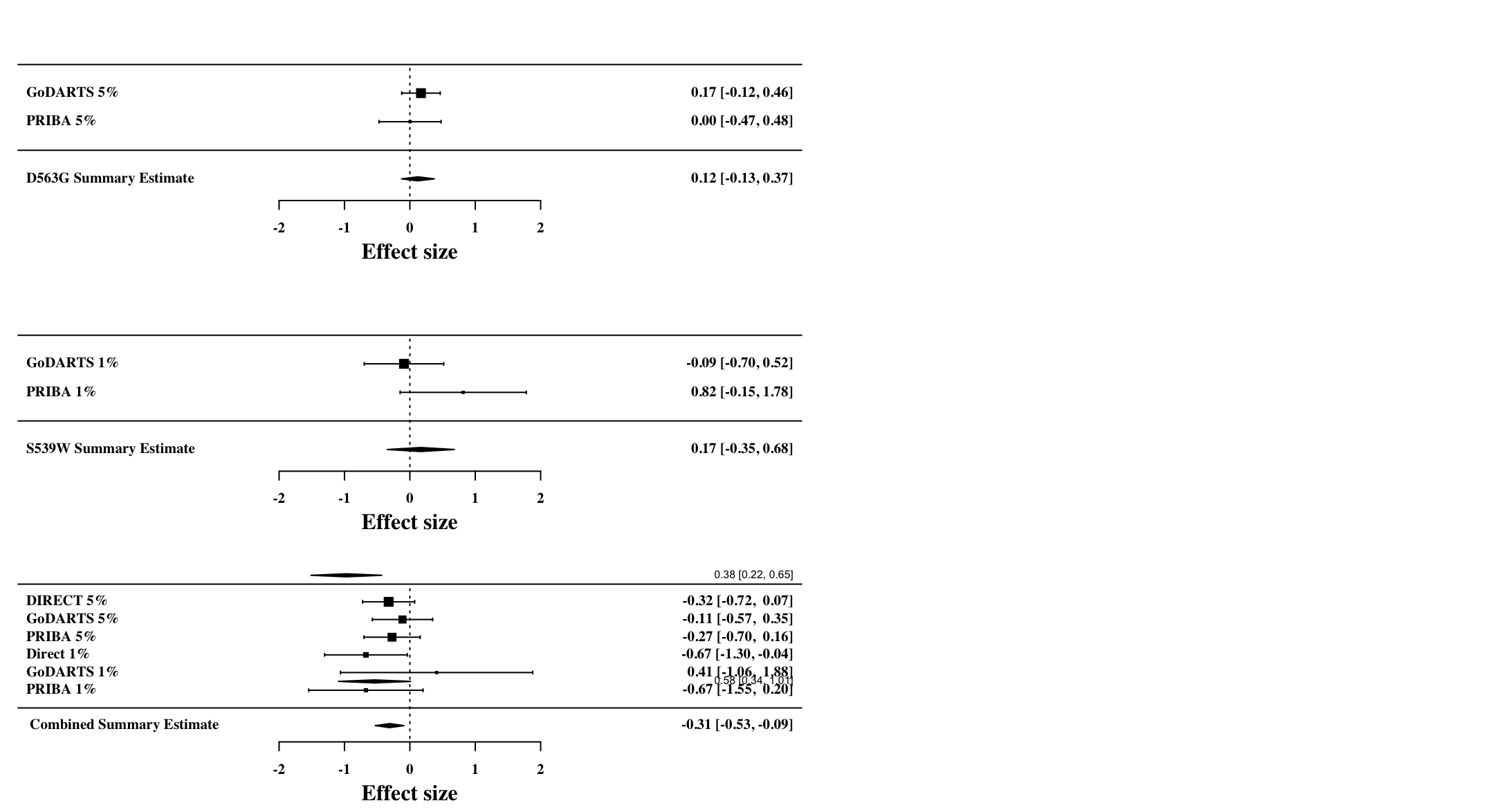

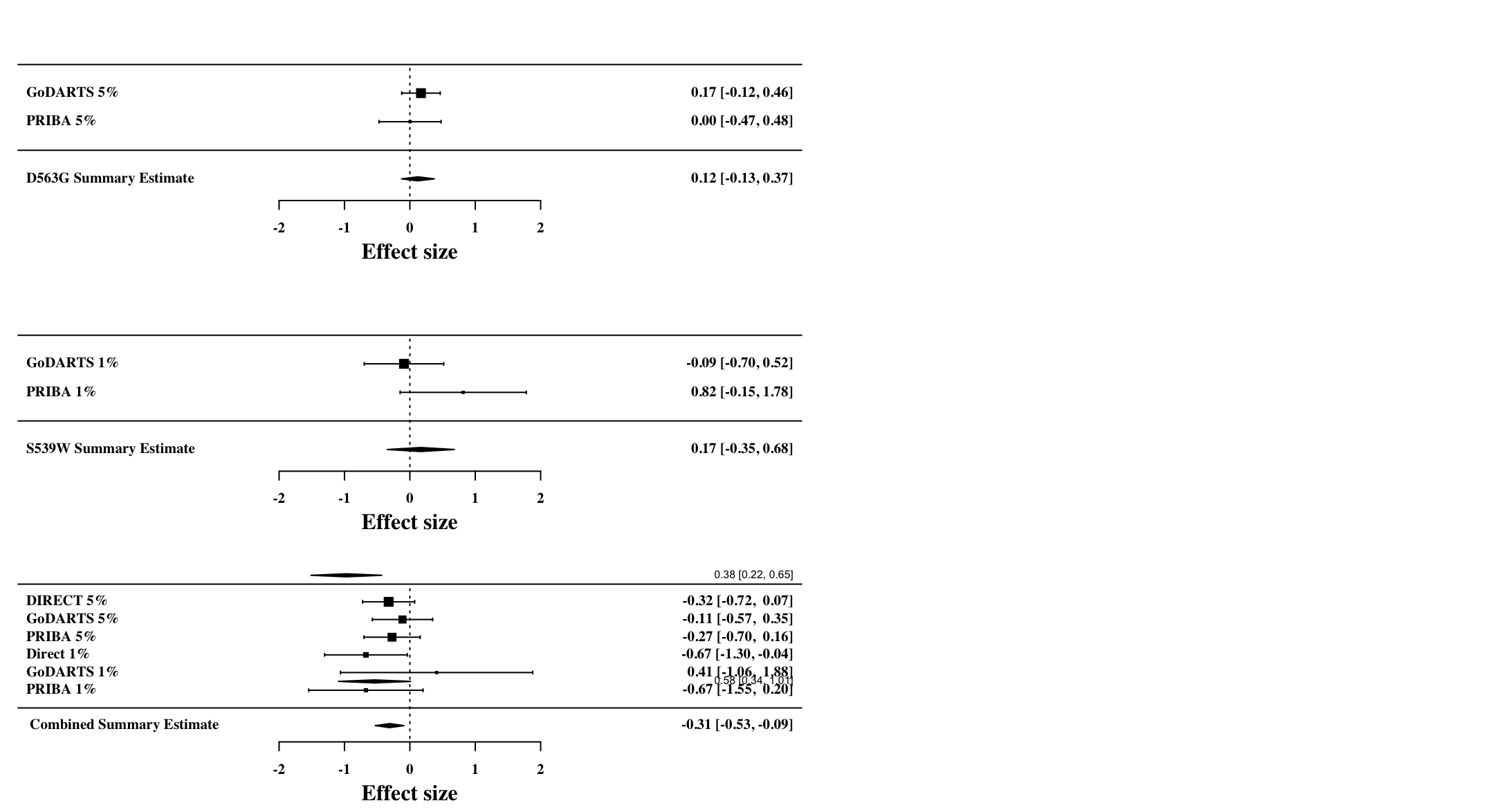


**Meta-analysis of the effect of carrying D536G and S539W on response to DPP-IVi therapy**

Figure 3.1 demonstrates the effect of carrying D536G and S539W on treatment response to DPP-IVi. Each cohort is displayed separately and the effect size is indicated by the location of a solid box with the 95% CI displayed either side. The line of no effect is indicated by a vertical dotted line. The summary estimate of the effect of each allele is displayed below the individual cohort summaries and is indicated by a solid black diamond with the centre of the diamond indicating the summary estimate and the lateral points the 95%CI.

**GSK Harmony Pharmacogenetics**

| rs78408340C>G  n= 1292  MAF= 0.003 | *Covariate Adjustment* | *Beta* | *SE* | P |
| --- | --- | --- | --- | --- |
|  | No | 0.30902 | 0.21241 | 0.146 |
|  | Yes* | 0.282551 | 0.184418 | 0.1257 |

| rs35658696A>G  n=1292  MAF= 0.043 | *Covariate Adjustment* | *Beta* | SE | P |
| --- | --- | --- | --- | --- |
|  | No | -0.03222 | 0.08035 | 0.689 |
|  | Yes* | -0.043090 | 0.069991 | 0.5382 |

*Baseline HbA1c + BMI fall + Duration Diabetes + Age + gender + study site.

**EXSCEL Study Demographics**

| Gender (N, %) | Male (969, 64.34%) |
| --- | --- |
|  | Female (537, 35.66%) |
| Age (N, %) | 20 < x <= 25 (1, 0.07%) |
|  | 25 < x <= 30 (2, 0.13%) |
|  | 30 < x <= 35 (1, 0.07%) |
|  | 35 < x <= 40 (12, 0.80%) |
|  | 40 < x <= 45 (39, 2.59%) |
|  | 45 < x <= 50 (68, 4.52%) |
|  | 50 < x <= 55 (151, 10.03%) |
|  | 55 < x <= 60 (267, 17.73%) |
|  | 60 < x <= 65 (315, 20.92%) |
|  | 65 < x <= 70 (304, 20.19%) |
|  | 70 < x <= 75 (203, 13.48%) |
|  | 75 < x <= 80 (98, 6.51%) |
|  | 80 < x <= 85 (38, 2.52%) |
|  | 85 < x <= 90 (7, 0.46%) |
| BMI (N, %) | 0 < x <= 19 (36, 2.39%) |
|  | 19 < x <= 25 (43, 2.86%) |
|  | 25 < x <= 30 (293, 19.46%) |
|  | 30 < x <= 80 (1134, 75.30%) |

| rs35658696A>G  N=1506  MAF= 0.043 | *Beta* | 95%CI | P |
| --- | --- | --- | --- |
|  | 0.037 | -{0.213,0.287] | P=0.77 |

**Section 4: Study Plan for prospective recruit-by-genotype study**

**Study Title:**  Defining the physiological mechanisms of risk genes for hyperglycaemia, insulin resistance and type 2 diabetes - *Peptidylglycine alpha-amidating mono-oxygenase (PAM)*

**Internal Reference Number / Short title:** DIVA – Diabetes Variants Study - *Peptidylglycine alpha-amidating mono-oxygenase (PAM)*

**Ethics Ref:** 15/SC/0072

| **Chief Investigator:** | Prof Fredrik Karpe, OCDEM, RDM, University of Oxford |
| --- | --- |
| **Investigators:** | Prof Mark I McCarthy, Dr Anna L Gloyn, Prof Patrik Rorsman |
| **Sponsor:** | University of Oxford |
| **Funder:** | Medical Research Council |

# 1.0 Background and Rationale

Treatment of T2D focuses on lowering glycemic exposure with lifestyle and pharmacological interventions (Inzucchi, Bergenstal et al. 2012). Despite the availability of multiple medications to lower HbA1c, only 53% of individuals with T2D reach the glycemic target (HbA1c <7%) (Stark Casagrande, Fradkin et al. 2013, Dawed, Zhou et al. 2016). Inter-individual response to pharmacological interventions is high and delay to treatment with the optimal medication contributes to a greater overall glycemic exposure and development of complications (Holman, Paul et al. 2008, Dawed, Zhou et al. 2016). Currently, T2D treatment algorithms suggest clinical equipoise between the six second line agents (Inzucchi, Bergenstal et al. 2012). Choice of second line agent tends to be driven by clinician preference, side effect profile and co-morbid conditions. There is potential to improve medication selection through “precision medicine” where genetic markers are used to indicate whether a patient is more or less likely to respond to a medication.

Two independent coding alleles in *peptidylglycine alpha-amidating mono-oxygenase (PAM)* (p.S539W, rs78408340, minor allele frequency (MAF) ~1%, OR: 1.47 and p.D563G, rs35658696, MAF ~5%, OR: 1.23) alter T2D risk and beta-cell function (Voight, Scott et al. 2010, Huyghe, Jackson et al. 2013). The *PAM* gene encodes the PAM protein, which is a required enzyme for amidation (Eipper, Stoffers et al. 1992, Prigge, Mains et al. 2000). The conversion of C-terminal glycine residue to an amide group (amidation) is required for full biological activity of many hormones, including some that are known to regulate blood glucose concentration (e.g. gastrin and cholecsystokinin) (Rehfeld and Stadil 1973, Eipper, Stoffers et al. 1992, Czyzyk, Ning et al. 2005, Steinert, Feinle-Bisset et al. 2017). Recently studies have shown *in vitro* that PAM deficiency in pancreatic beta-cells results in reduced insulin content and altered dynamics of insulin secretion (Thomsen, Raimondo et al. 2018). Glucagon like peptide 1 (GLP-1), a second line treatment for T2D, is itself amidated and interacts with a number of other amidated peptides (Inzucchi, Bergenstal et al. 2012).

# 2.0 Hypothesis:

We hypothesised that individuals carrying T2D-risk alleles in PAM would have reduced serum amidation activity and altered GLP-1 concentration which could influence glucose homeostasis. Altered GLP-1 plasma levels or GLP-1 response in carriers of PAM T2D-risk alleles would have implications for the efficacy of two commonly prescribed medications for type 2 diabetes, GLP-1 Receptor Agonists (GLP-1RA) and dipeptidyl-peptidase 4 inhibitors (DPP-IVi). As 10% of individuals carry a T2D-risk variant in the *PAM* gene this could impact medication choice for many individuals with T2D.

# 3.0 Outcomes:

**Primary Outcome:** The incretin effect expressed as a percentage

**Secondary Outcomes:** Insulin profile during OGTT, glucose proile during OGTT, Amidated GLP-1, Unamidated GLP-1, Serum PAM enzyme activity

# 4.0 Ethics Approval and Clinical Trials Registry

This study was reviewed and approved by the NRES Oxford B Research Ethics Committee (15/SC/0072).

The study was registered on the clinicaltrials.gov trial registry (NCT02723110).

# 5.0 Study Design and Recruitment

Twenty heterozygous carriers of the S539W allele and 20 age, gender and BMI matched non-carriers were recruited into a double-blind, observational, recruit-by-genotype study. Volunteers were recruited from the OBB.

| **Inclusion Criteria** | **Exclusion Criteria** |
| --- | --- |
| Age: 30-65 years | Previous resection or banding of the stomach, small bowel or large bowel |
| Non-diabetic (fasting BGL<5.5mmol/L) | On medication effecting insulin secretion, or glucose homeostasis |
| Enrolled in the OBB | Carrier of the rs35658696 *PAM* variant |
| rs78408340 heterozygote, or matched control | Anaemia |
| Able to provide informed consent | Recent blood donation |

**Table 1. Inclusion and exclusion criteria**

# 6.0 Volunteer Identification and Recruitment

The study population is from the Oxford Biobank (OBB) (Oxford C 08/H0606/107+5, NRES 136602 part of the NIHR Bioresource) which is a random collection of 30-50 year old healthy men and women living in Oxfordshire who have participated in a screening project including donation of DNA and provision of informed consent to be recalled. OBB participants will be invited to take part based on their genotype (*PAM* gene variant carrier status) along with age, sex and BMI matched non-carrier controls.

Carriers of S539W allele were identified in the OBB in using an allelic discrimination assay. Non-carriers were also recruited from the OBB. Three to five matched non-carriers were invited to the study concurrently with each carrier to expedite recruitment. All participants were screened for the other T2D risk allele in *PAM* (D536G). If a volunteer carried the D536G allele, they were excluded from the study.

# 7.0 Study Protocol

Carriers and matched non-carriers of S539W underwent an oral glucose tolerance test (OGTT) and isoglycaemic clamp (**Figure 1**) on separate days. The two study days were separated by no less than 7 days.

On Study Day 1, participants attended the CRU following a 12 hour fast. A retrograde cannula was inserted into the dorsum of the hand for blood sampling. The hand was placed in a “hotbox” to arterialise venous blood (Nauck, Liess et al. 1992) (figure 4.3). A 75g oral glucose load was administered over 5 minutes. Blood was sampled every 5 minutes for 240 minutes to determine blood glucose concentration.

On Study Day 2, participants attended the CRU following a 12 hour fast. A retrograde cannula was inserted into the dorsum of the hand for blood sampling. The hand was again placed in a “hotbox” to arterialise the venous blood. An anterograde cannula was placed in the antecubital fossa of the contralateral to allow intravenous glucose delivery. A variable intravenous infusion of 20% glucose was administered over 240 minutes to reproduce the glucose profile of the OGTT.

At 10 time points on both study days samples were drawn (15ml) and stored in 3 different tube types: potassium EDTA tubes with dipeptidyl peptidase IV (DPP-IV) inhibitor, lithium-heparin tubes and serum tubes. Potassium EDTA and lithium-heparin tubes were placed on ice and immediately centrifuged, then aliquoted on ice, and stored at -80°C. The serum tubes were left at room temperature for approximately 30 min to facilitate clotting before centrifugation and storage at -80°C.

**Figure 1 Study Protocol: Frequently sampled OGTT and isoglycaemic clamp.**

Blood samples to be collected at -15, 0, 15, 30, 45, 60, 90, 120, 180, 240min.

**Confirmation of genotype**

To ensure that volunteers of the correct genotype were included, the leucocyte rich layer or “buffy coat” was stored and DNA extracted following the completion of recruitment. Genotypes of samples collected from the participants were cross-referenced with those generated from DNA stored in the OBB.

# 8.0 Sample size calculation

Given the novel nature of this study, establishing the expected effect size was difficult. Due to uncertainty regarding the effect size in the primary outcome (incretin effect), an interim analysis at 20 v 20 volunteers will be performed. An adaptive design approach will be utilised with stringent criteria for stopping for futility or clear effect (Table 2). It was prospectively determined that if a trend towards a significant outcome was detected, a further 10 matched pairs would be included. The decision to continue to recruit further volunteers was based on the beta of the primary outcome observed in the first 20 pairs (Table 2). This approach was used to allow an interim analysis and minimise the adjustment for multiple testing [protocol developed through collaboration with Dr Christopher Jennison (Biostatistician, University of Bath)].

|  | Stop and reject null hypothesis | Effect size for rejecting null hypothesis | Stop and accept null hypothesis | Effect size for rejecting |
| --- | --- | --- | --- | --- |
| Stage 1: (20v20) | Z > 2.18 | >15.2% | Z > 1.69 | -2.2% |
| Stage 2: (10v10) | Z < -0.32 | >11.8% | Z > 1.69 | <11.8% |

**Table 2. Criteria for accepting or rejecting the null hypothesis at both stages of clinical study.**

The criteria for stopping due to futility or rejection of the null hypothesis are based on a group sequential test and use the standard deviation of the incretin effect (7%). The Z boundary has been calculated in statistical program ‘R’ using a group sequential test model and converted in to an effect size. Type I error rate for group sequential t-test = 0.05. This provided a 90% chance of detecting a difference of 10% with an alpha 5% (estimated SD of 10%) in incretin effect at the first stopping point.

# 9.0 Biochemical Analysis

Blood samples will be collected at 10 time points during the OGTT. Prior to blood sampling, 5 ml of blood will be drawn from the intravenous cannula to prevent dilution from previous flushes. To minimise total blood loss, this 5 ml of blood will be returned to the patient following collection of the sample. The sample will be drawn and placed into either a serum, lithium heparin or plasma EDTA tube. Plasma tubes will be stored frozen at -20° C until blood collection.

In the case of plasma, samples will be collected in chilled tubes and transferred on ice to be spun immediately. Serum will be stored at room temperature in collection tubes for 30 minutes to allow blood to clot and will then be centrifuged. Samples will be centrifuged for 15 minutes at 3000 RPM and 4° C. Plasma and serum will then aliquoted and stored at -80° C.

**DNA extraction**

Following centrifugation of the EDTA tubes, the leuckocyte rich “buffy coat” will be removed and stored at -80C. On completion of the study, genotypes will be determined by extracting DNA using the Maxwell 16 (Promega Corporation, Madison, USA). This DNA will then be genotyped using allelic discrimination genotyping and compared to genotypes generated from the stored OBB DNA samples.

**Amidation activity**

The amidation activity will be measured in all participants to determine if there is any alteration in serum amidation activity. Fifty percent recombinant PAM was used as an internal control to allow comparison between assay batches.

**Insulin**

Insulin concentration will be measured using a commercial radioimmunoassay kit, the Human Specific Insulin RIA Kit (EMD Milipore, Billerica, USA).

**Glucose**

Glucose will be measured on the day of the study. Glucose was measured using Ilab 650 Analyser (Instrumentation Laboratory Ltd, Warrington, UK). Realtime point of care glucose measurement on each blood draw to facilitate adjustment of the variable glucose infusion using The HemoCue Glucose 201+ System (HemoCue, Ängelholm, Sweden).

**GLP-1 (7-36)amide and GLP-1 (7-37)gly measurment**

Plasma samples will be sent to the Holst lab in Denmark for measurement of GLP-1 with in-house assays, which are specific for the amidation state. Samples will be sent for all 10 time points of the OGTT (Wettergren, Pridal et al. 1998).

**Gastrin and CCK Measurement**

Gastrin and CCK will be sent to the Rehfeld lab in Denmark who possess a specific assay for detection of the amidated and glycine extended forms of both peptides. Due to the limits of sample availability and the likelihoods that if a difference was present between carriers and non-carriers CCK and gastrin will only be measure at baseline and peak postprandial concentration. The reported peak concentration for gastin and CCK are 30 and 60 min respectively (Rehfeld and Stadil 1973, Rehfeld 1998, Steinert, Feinle-Bisset et al. 2017).

**TSH**

Fasting serum samples will be sent to the clinical biochemistry laboratory at the John Radcliffe Hospital.

**Incretin Effect**

The incretin effect will be calculated using the two methods routinely used in the literature. Firstly, the incretin effect will be measured using gastrointestinal mediated glucose disposal. This method calculates the incretin effect by measuring the difference in the amount of intravenous glucose and oral glucose required to produce the same glucose profile. The rational underpinning of this method is that additional glucose disposed of during an oral glucose load is due to incretin hormones. Measurement of insulin to determine the difference in the insulin AUC between OGTT and matched isoglycaemic infusion will also be performed.

The incretin effect will be calculated using the following formulae;

1. 100 x (oral glucose consumed - IV glucose administered) ÷ oral glucose consumed
2. 100 x (insulin AUC OGTT - insulin AUC matched isoglycaemic clamp) ÷ insulin AUC OGTT

# 10.0 Informed Consent

Informed consent will be obtained by a suitably qualified and experienced research nurse, or a study doctor who has been authorised to do so by the CI/PI. The original consent will be retained in the CRF, all participants will receive a copy.

The participant will have received the PIS several days before the scheduled initial visit.

At the initial visit the participant will also receive a verbal description of the exact nature of the study; what their participation will involve (the implications and constraints of the protocol) the known side effects and any risks involved in taking part. It will be clearly stated that the participant is free to withdraw from the study at any time for any reason without prejudice to future care, and with no obligation to give the reason for withdrawal. Written Informed Consent will then be obtained by means of participant dated signature and dated signature of the person who presented and obtained the Informed Consent.

## Screening and Eligibility Assessment

Screening and eligibility takes place at two steps. First before recruitment with help of the information held within the OBB, and second, at the initial visit where additional information relevant to the exclusion criteria might become apparent. Participants will also be screened for anaemia on the first blood sample drawn. This will be checked on a point of care machine and the participant withdrawn if this reading demonstrates anaemia.

##

# 11.0 Blinding and code-breaking

The study is double blind through an internal blinding procedure. Gene carriers and controls are identified by one of the researchers within the OBB team who does not have access to names and contact details. This researcher assembles suitable participants to be invited (by their allocated OBB number). Once the OBB number is linked to the name and contact detail (the gene, the genotype and the other participant details are unknown to the recruitment nurse at this stage), the recruitment starts. The participants have provided informed consent in their first screening visit as OBB participants to remain unaware of their gene carrier status.

During the course of a study when data is collected, the ID of the participant is their study number. Genotype details therefore will never go back to the researcher who eventually unblinds the genotype information (A or B) once all analyses are completed.

**References:**

Czyzyk, T. A., Y. Ning, M.-S. Hsu, B. Peng, R. E. Mains, B. A. Eipper and J. E. Pintar (2005). "Deletion of peptide amidation enzymatic activity leads to edema and embryonic lethality in the mouse." Developmental Biology **287**(2): 301-313.

Dawed, A. Y., K. Zhou and E. R. Pearson (2016). "Pharmacogenetics in type 2 diabetes: influence on response to oral hypoglycemic agents." Pharmgenomics Pers Med **9**: 17-29.

Eipper, B. A., D. A. Stoffers and R. E. Mains (1992). "The biosynthesis of neuropeptides: peptide alpha-amidation." Annu Rev Neurosci **15**: 57-85.

Holman, R. R., S. K. Paul, M. A. Bethel, D. R. Matthews and H. A. Neil (2008). "10-year follow-up of intensive glucose control in type 2 diabetes." N Engl J Med **359**(15): 1577-1589.

Huyghe, J. R., A. U. Jackson, M. P. Fogarty, M. L. Buchkovich, A. Stancakova, H. M. Stringham, X. Sim, L. Yang, C. Fuchsberger, H. Cederberg, P. S. Chines, T. M. Teslovich, J. M. Romm, H. Ling, I. McMullen, R. Ingersoll, E. W. Pugh, K. F. Doheny, B. M. Neale, M. J. Daly, J. Kuusisto, L. J. Scott, H. M. Kang, F. S. Collins, G. R. Abecasis, R. M. Watanabe, M. Boehnke, M. Laakso and K. L. Mohlke (2013). "Exome array analysis identifies new loci and low-frequency variants influencing insulin processing and secretion." Nature genetics **45**(2): 197-201.

Inzucchi, S. E., R. M. Bergenstal, J. B. Buse, M. Diamant, E. Ferrannini, M. Nauck, A. L. Peters, A. Tsapas, R. Wender and D. R. Matthews (2012). "Management of hyperglycemia in type 2 diabetes: a patient-centered approach: position statement of the American Diabetes Association (ADA) and the European Association for the Study of Diabetes (EASD)." Diabetes Care **35**(6): 1364-1379.

Nauck, M. A., H. Liess, E. G. Siegel, P. D. Niedmann and W. Creutzfeldt (1992). "Critical evaluation of the 'heated-hand-technique' for obtaining 'arterialized' venous blood: incomplete arterialization and alterations in glucagon responses." Clin Physiol **12**(5): 537-552.

Prigge, S. T., R. E. Mains, B. A. Eipper and L. M. Amzel (2000). "New insights into copper monooxygenases and peptide amidation: structure, mechanism and function." Cellular and molecular life sciences : CMLS **57**(8-9): 1236-1259.

Rehfeld, J. F. (1998). "Accurate measurement of cholecystokinin in plasma." Clin Chem **44**(5): 991-1001.

Rehfeld, J. F. and F. Stadil (1973). "The effect of gastrin on basal- and glucose-stimulated insulin secretion in man." J Clin Invest **52**(6): 1415-1426.

Stark Casagrande, S., J. E. Fradkin, S. H. Saydah, K. F. Rust and C. C. Cowie (2013). "The prevalence of meeting A1C, blood pressure, and LDL goals among people with diabetes, 1988-2010." Diabetes Care **36**(8): 2271-2279.

Steinert, R. E., C. Feinle-Bisset, L. Asarian, M. Horowitz, C. Beglinger and N. Geary (2017). "Ghrelin, CCK, GLP-1, and PYY(3-36): Secretory Controls and Physiological Roles in Eating and Glycemia in Health, Obesity, and After RYGB." Physiol Rev **97**(1): 411-463.

Thomsen, S. K., A. Raimondo, B. Hastoy, S. Sengupta, X. Q. Dai, A. Bautista, J. Censin, A. J. Payne, M. M. Umapathysivam, A. F. Spigelman, A. Barrett, C. J. Groves, N. L. Beer, J. E. Manning Fox, M. I. McCarthy, A. Clark, A. Mahajan, P. Rorsman, P. E. MacDonald and A. L. Gloyn (2018). "Type 2 diabetes risk alleles in PAM impact insulin release from human pancreatic beta-cells." Nat Genet **50**(8): 1122-1131.

Voight, B. F., L. J. Scott, V. Steinthorsdottir, A. P. Morris, C. Dina, R. P. Welch, E. Zeggini, C. Huth, Y. S. Aulchenko, G. Thorleifsson, L. J. McCulloch, T. Ferreira, H. Grallert, N. Amin, G. Wu, C. J. Willer, S. Raychaudhuri, S. A. McCarroll, C. Langenberg, O. M. Hofmann, J. Dupuis, L. Qi, A. V. Segre, M. van Hoek, P. Navarro, K. Ardlie, B. Balkau, R. Benediktsson, A. J. Bennett, R. Blagieva, E. Boerwinkle, L. L. Bonnycastle, K. Bengtsson Bostrom, B. Bravenboer, S. Bumpstead, N. P. Burtt, G. Charpentier, P. S. Chines, M. Cornelis, D. J. Couper, G. Crawford, A. S. Doney, K. S. Elliott, A. L. Elliott, M. R. Erdos, C. S. Fox, C. S. Franklin, M. Ganser, C. Gieger, N. Grarup, T. Green, S. Griffin, C. J. Groves, C. Guiducci, S. Hadjadj, N. Hassanali, C. Herder, B. Isomaa, A. U. Jackson, P. R. Johnson, T. Jorgensen, W. H. Kao, N. Klopp, A. Kong, P. Kraft, J. Kuusisto, T. Lauritzen, M. Li, A. Lieverse, C. M. Lindgren, V. Lyssenko, M. Marre, T. Meitinger, K. Midthjell, M. A. Morken, N. Narisu, P. Nilsson, K. R. Owen, F. Payne, J. R. Perry, A. K. Petersen, C. Platou, C. Proenca, I. Prokopenko, W. Rathmann, N. W. Rayner, N. R. Robertson, G. Rocheleau, M. Roden, M. J. Sampson, R. Saxena, B. M. Shields, P. Shrader, G. Sigurdsson, T. Sparso, K. Strassburger, H. M. Stringham, Q. Sun, A. J. Swift, B. Thorand, J. Tichet, T. Tuomi, R. M. van Dam, T. W. van Haeften, T. van Herpt, J. V. van Vliet-Ostaptchouk, G. B. Walters, M. N. Weedon, C. Wijmenga, J. Witteman, R. N. Bergman, S. Cauchi, F. S. Collins, A. L. Gloyn, U. Gyllensten, T. Hansen, W. A. Hide, G. A. Hitman, A. Hofman, D. J. Hunter, K. Hveem, M. Laakso, K. L. Mohlke, A. D. Morris, C. N. Palmer, P. P. Pramstaller, I. Rudan, E. Sijbrands, L. D. Stein, J. Tuomilehto, A. Uitterlinden, M. Walker, N. J. Wareham, R. M. Watanabe, G. R. Abecasis, B. O. Boehm, H. Campbell, M. J. Daly, A. T. Hattersley, F. B. Hu, J. B. Meigs, J. S. Pankow, O. Pedersen, H. E. Wichmann, I. Barroso, J. C. Florez, T. M. Frayling, L. Groop, R. Sladek, U. Thorsteinsdottir, J. F. Wilson, T. Illig, P. Froguel, C. M. van Duijn, K. Stefansson, D. Altshuler, M. Boehnke, M. I. McCarthy, M. investigators and G. Consortium (2010). "Twelve type 2 diabetes susceptibility loci identified through large-scale association analysis." Nat Genet **42**(7): 579-589.

Wettergren, A., L. Pridal, M. Wojdemann and J. J. Holst (1998). "Amidated and non-amidated glucagon-like peptide-1 (GLP-1): non-pancreatic effects (cephalic phase acid secretion) and stability in plasma in humans." Regul Pept **77**(1-3): 83-87.
