# Supplementary material for: Type 2 diabetes risk alleles in peptidyl-glycine alpha-amidating monooxygenase influence GLP-1 levels and response to GLP-1 receptor agonists
Source: Genome Med. 2026 Mar 29;18:40. doi: 10.1186/s13073-026-01630-0 (PMC13072570; doi:10.1186/s13073-026-01630-0)

# Figure S1: Generation of *Pam^fl/fl^* (WT) mice and validation of efficient

**loss of PAM in PamKO mouse tissues**

**a,** Schematic representation of mouse targeting strategy, and creation of *Pam^fl/fl^* allele. **b,c,** Pam expression in isolated pancreatic islets (**b**) and pituitary (**c**) of PamKO and WT littermate control mice (n = 2 and 3, respectively). **d,** PCR analysis of recombined *Pam^fl/fl^* allele in liver, stomach or duodenum of PamKO mice and WT littermate controls. Location of PCR primers is depicted in green on panel **a**. **e,** HPLC absorbance traces at 220 nm of amidation assay results from pituitary extracts of PamKO and WT control mice. The plots show amidation assay substrate (Dansyl-YVG), intermediate (Dansyl-YVG-COOH) and product (Dansyl-YV-NH2) at different time points after the start of the enzymatic reaction. **f,** blood glucose levels of 10 week-old mice of indicated genotype, 5 weeks after tamoxifen treatment (n=5,5). **g,** body weights of mice of indicated genotype and sex. Data are presented as mean ± SD. Two tailed *t* test (**f**) and 2-way repeated measures ANOVA with Sidak’s multiple comparisons test (**g**), **P* < 0.05, ***P* < 0.01, ****P* < 0.005, *****P* < 0.001.


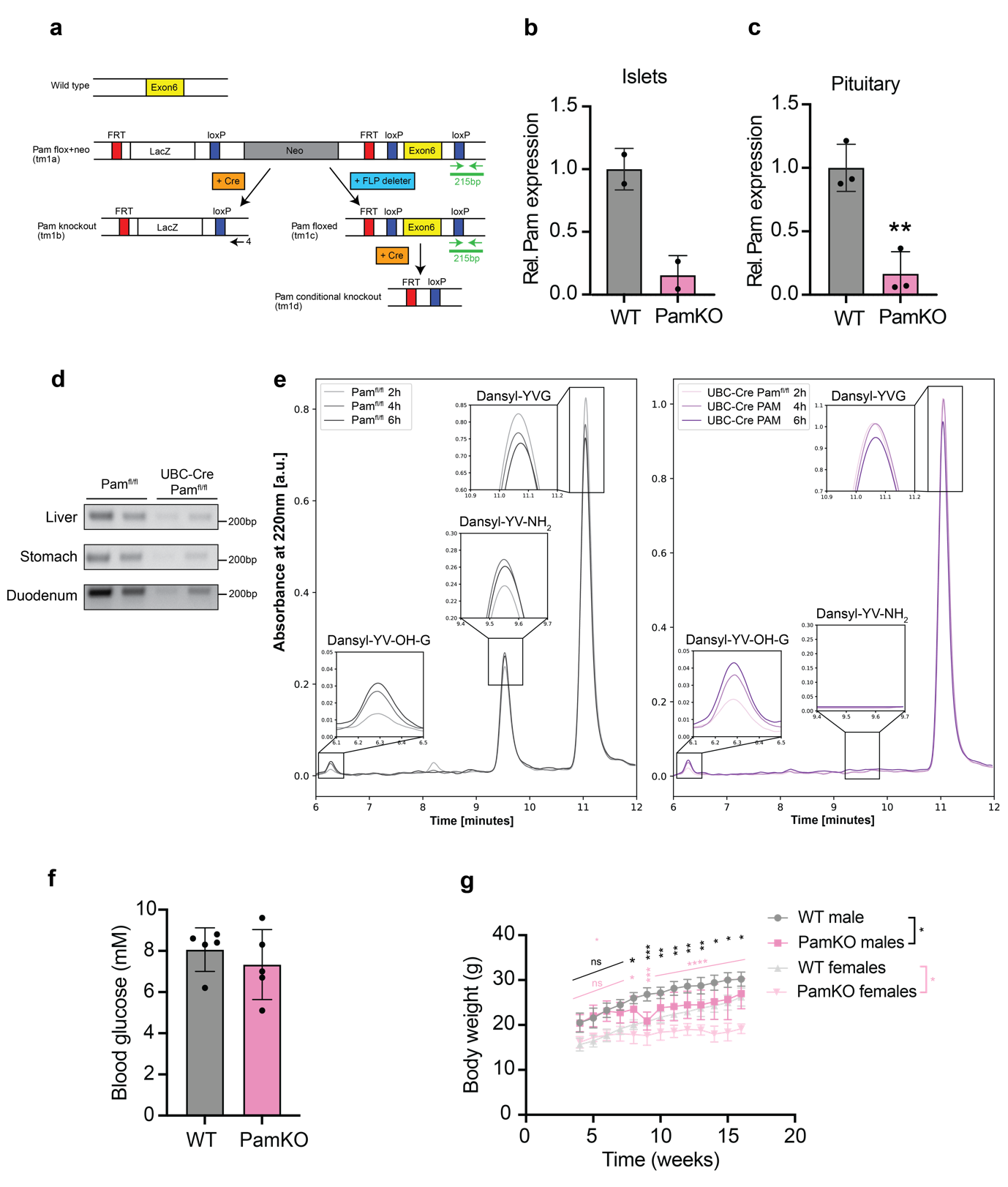


**Figure S2: GLP-1 (7-36 amide) and GLP-1 (7-37 Gly-extended) are both biologically active and equipotent**

**a,** Fasting GLP-1 (7-36) NH_2_ and GLP-1 7-37-Gly concentrations in stored plasma from the Oxford Biobank, and the ratio between them, in heterozygous carriers of p.S539W and matched non-carriers.

**b,** Receptor binding affinity assay in HEK293A cells transfected with hGLP-1R, in competition with ^125^I-GLP-1(7-36 amide) for both GLP-1(7-36)NH_2_ and GLP-1 (7-37Gly). Gamma radiation of bound ^125^I-GLP-1(7-36 amide) is represented at varying concentrations of unlabeled GLP-1(7-36)NH_2_ and GLP-1(7-37Gly). **c,** Intracellular cAMP accumulation in HEK293A cells transfected with hGLP-1R and treated for 30 minutes with varying concentrations of GLP-1 (7-36)NH_2_ and GLP-1 (7-37Gly). **d,** Receptor internalization assay in HEK293A cells transfected with SNAP-hGLP-1R, labeled with the donor SNAP-Lumi4-Tb and acceptor fluorescein-O′-acetic acid, treated with varying concentrations of GLP-1(7-36)NH_2_ and GLP-1(7-37Gly) and donor/acceptor signals measured every 3 minutes**.** N=3. **e,** β-arrestin 2 recruitment in HEK293A transfected with hGLP-1R, Rluc8-Arr3-Sp2, mem-citrine and treated for 30 minutes with varying concentrations of GLP-1(7-36)NH_2_ and GLP-1(7-37Gly).


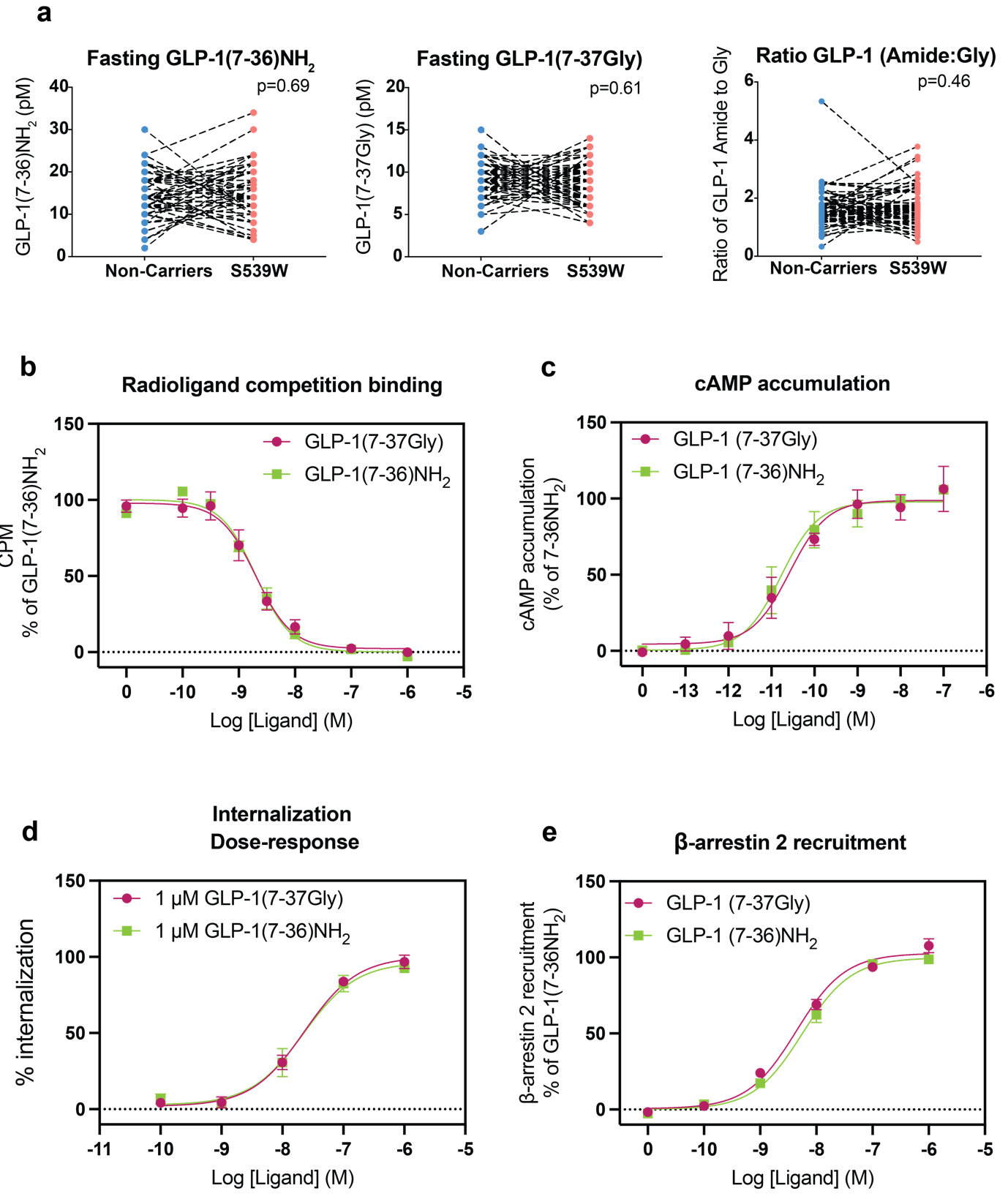


**Figure S3: GLP1 expression in tissues of PamKO and WT littermate control mice**

**a–c**, relative preglucagon (Gcg) mRNA expression in duodenum (**a**), ileum (**b**) and colon (**c**), (n=4). **d,e**, GLP1 peptide measurements in the duodenum (**d**) and jejunum (**e**) (n=7). **f**, Representative images (scale bar: 200 µm) (**f**) and quantification (n = 10 sections) (**g**) of PamKO and WT littermate control mice. Red, GLP1; blue, Hoechst. **h**, plasma DPP4 activity in PamKO and littermate control mice (n=5).


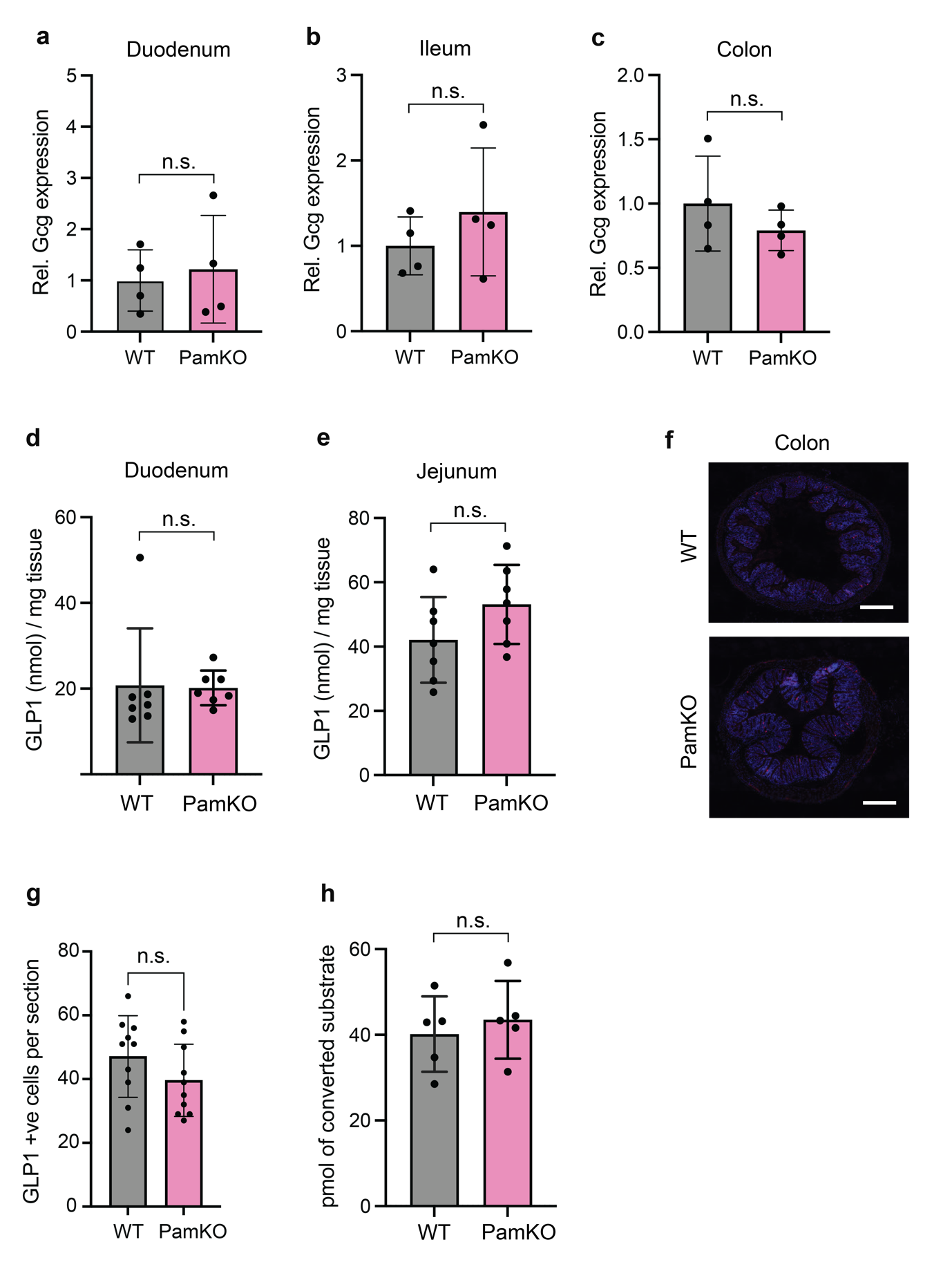


**Figure S4: Abundance of amidated hormones in PAM S539W heterozygote carriers is unchanged compared to non carriers**

**a,** Gastrin amide concentration before and 30 minutes after 75g OGTT. **b,** Glycine-extended (non-amidated) gastrin concentration before and 30 minutes after 75g OGTT. **c,** Cholecystokinin(CCK) amide concentration before and 60 minutes after 75g OGTT. **d,** Islet amyloid polypeptide (IAPP) amide concentration before and 90 minutes after 75g OGTT. **e,** Glycine-extended (non-amidated) islet amyloid polypeptide (IAPP) concentration before and 90 minutes after 75g OGTT


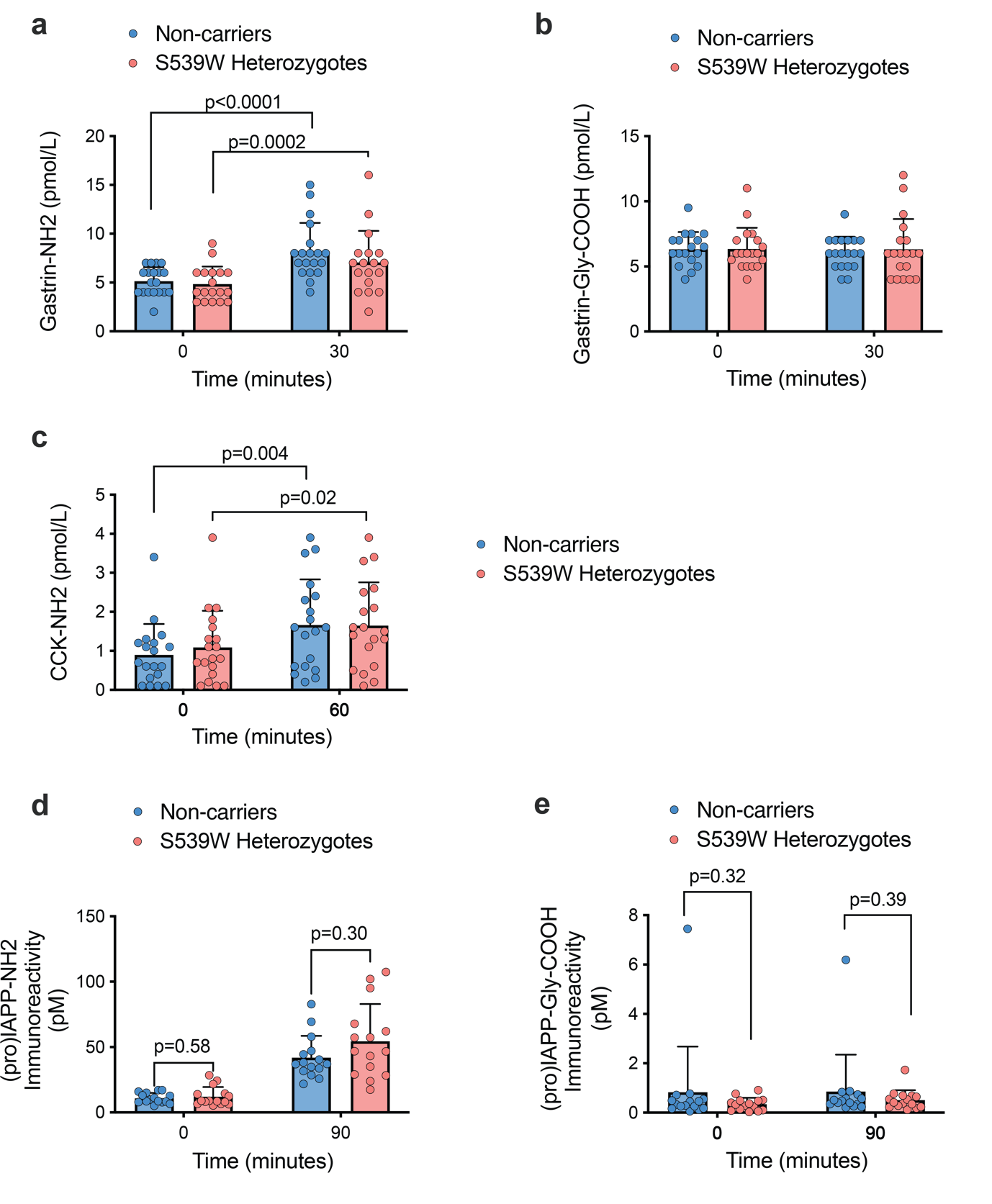


# Figure S5: Normal glucose tolerance, Glp1r expression but increased pyloric Grpr levels in PamKO compared to control mice

**a**, plasma insulin levels during oGTT in PamKO and WT littermate mice (n=7,7). **b**, Blood glucose levels during and oGTT *in Pdx-Cre* *Pam*^fl/fl^ and *Pam*^fl/fl^ mice (n=7,4). **c–h**, expression of Glp1r transcript levels in (**c**) pylorus (n=5,7), (**d**) kidneys (n=5,7), (**e**) hypothalamus (n=5,7), (**f**) cerebellum (n=5,6), (**g**) pituitary (n=5,7), (**h**) pancreatic islets (n=6,7) of PamKO and WT littermate mice. **i–j**, relative transcript levels of cholecystokinin B receptor (Cckbr) (**i**) and Gastrin releasing peptide receptor (Grpr)(**j**) in PamKO and WT littermate mice (n=6,7). Data are presented as mean ± SD. **P* < 0.05; no asterisk or n.s. indicates *P* > 0.05; two tailed *t* test.


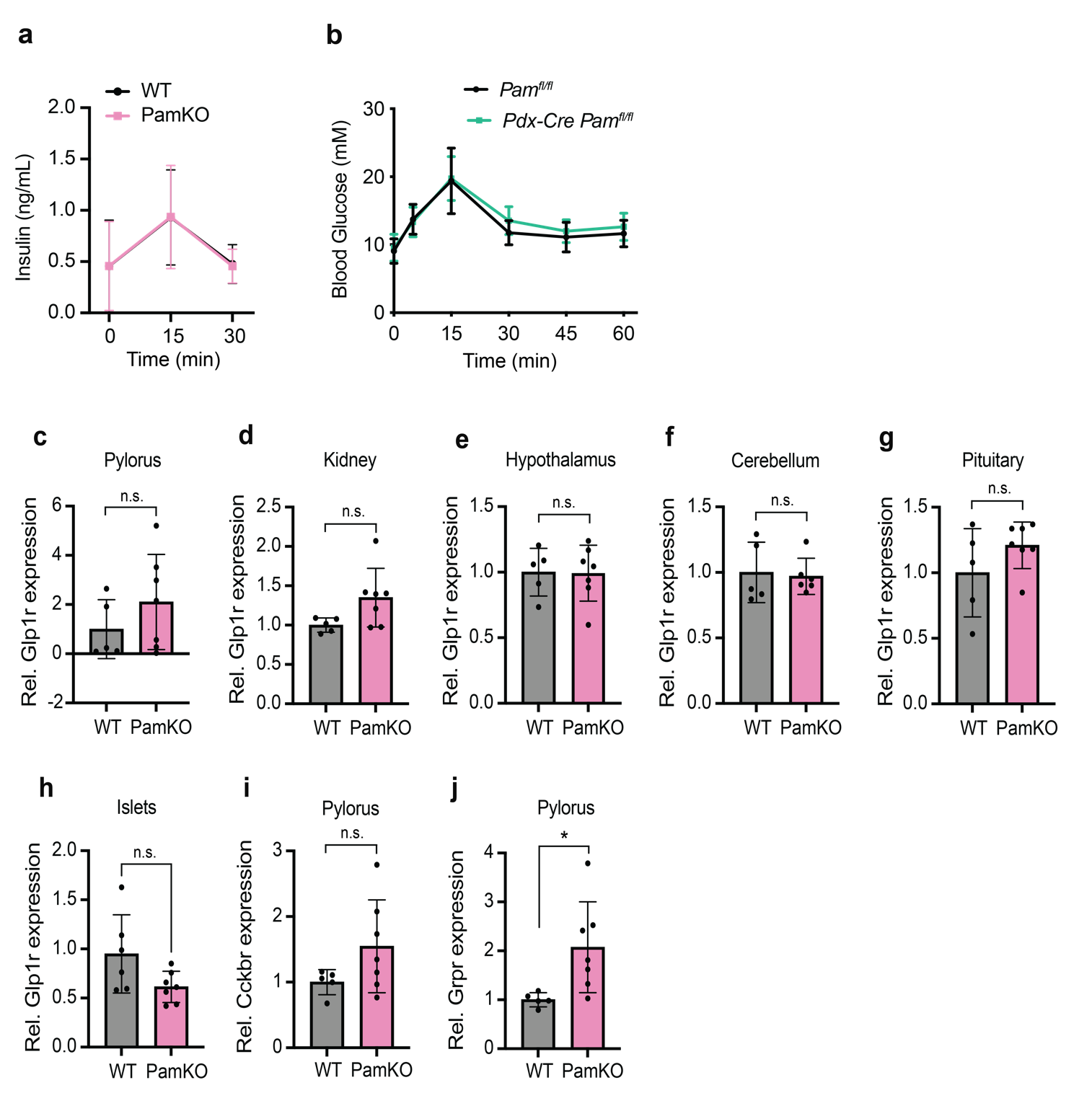


**Figure S6:** **Meta-analysis of the effect of carrying D536G and S539W on response to DPP-IVi therapy**

Figure demonstrates the effect of carrying D536G and S539W on treatment response to DPP-IVi. Each cohort is displayed separately and the effect size is indicated by the location of a solid box with the 95% CI displayed either side. The line of no effect is indicated by a vertical dotted line. The summary estimate of the effect of each allele is displayed below the individual cohort summaries and is indicated by a solid black diamond with the centre of the diamond indicating the summary estimate and the lateral points the 95%CI. Left of the vertical dotted line demonstrates reduced response, right of the vertical dotted line demonstrates greater response.


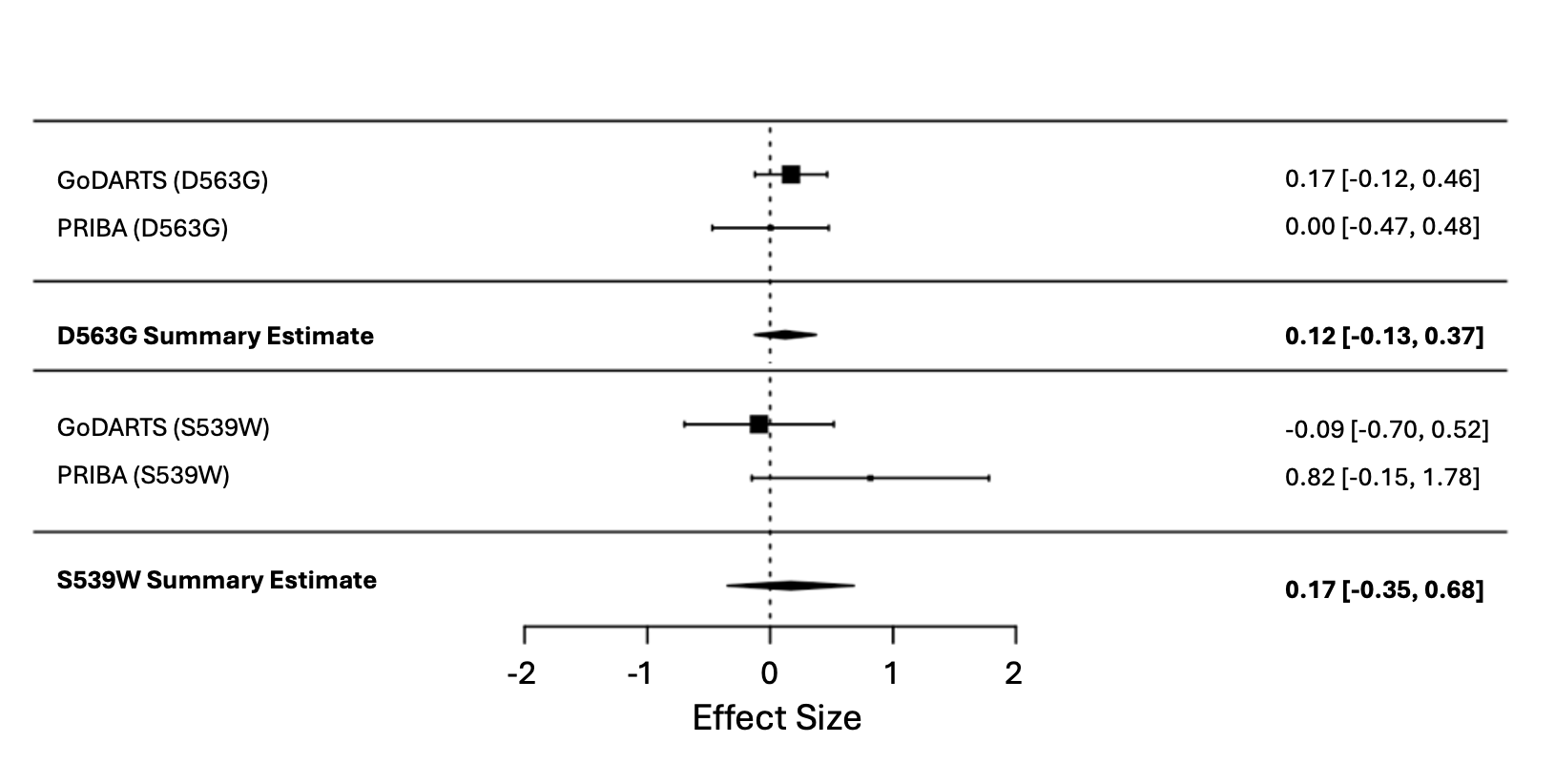


**Figure S7:** **Effect of PAM T2D risk alleles on treatment response in the GSK Harmony Study and agonist specific effects across cohorts**

A: Demonstrates a meta-analysis examining the impact of genotype on liraglutide treatment response in 983 individuals across the IMI-Direct, PRIBA, GoDARTS and HARMONY studies. Carriers of rs35658696 (p.D563G) had a reduced change in HbA1c at 6 months of 0.25% per allele.

B: Demonstrates the impact of genotype on response to albiglutide in the HARMONY study in 1073 individuals. Carriers of rs35658696 (p.D563G) or rs78408340 (p.S539W) did not change HbA1c response to albiglutide at 6 months.

C:Demonstrates a meta-analysis examining the impact of genotype on long acting agonist in carriers of rs35658696 (p.D563G) or rs78408340. There was no difference in HbA1c response to long acting agonists at 6 months.

D:Demonstrates a meta-analysis examining the impact of genotype on short acting agonist in carriers of rs35658696 (p.D563G) or rs78408340. The only short acting agonist included was exenatide. Carriers of rs78408340 (p.S539W) had reduced efficacy with a reduced change in HbA1c at 6months of 0.84% per allele. No difference was detected in carriers of rs35658696 (p.D563G).

In all figures, each cohort is displayed separately and the effect size is indicated by the location of a solid box with the 95% CI displayed either side. The line of no effect is indicated by a vertical dotted line. The summary estimate of the effect of each allele is displayed below the individual cohort summaries and is indicated by a solid black diamond with the centre of the diamond indicating the summary estimate and the lateral points the 95%CI. Left of the vertical dotted line demonstrates reduced response, right of the vertical dotted line demonstrates greater response.


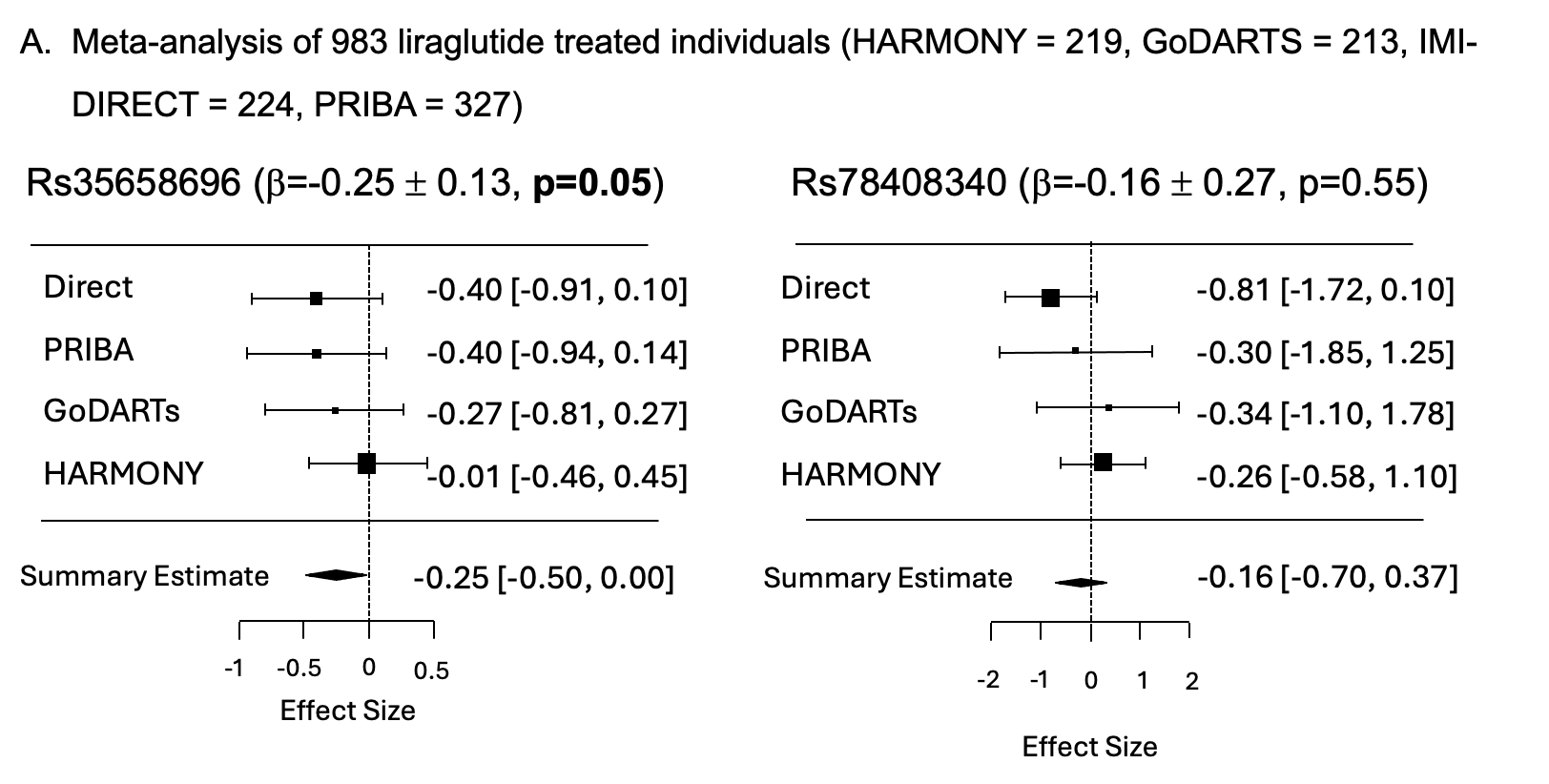


**
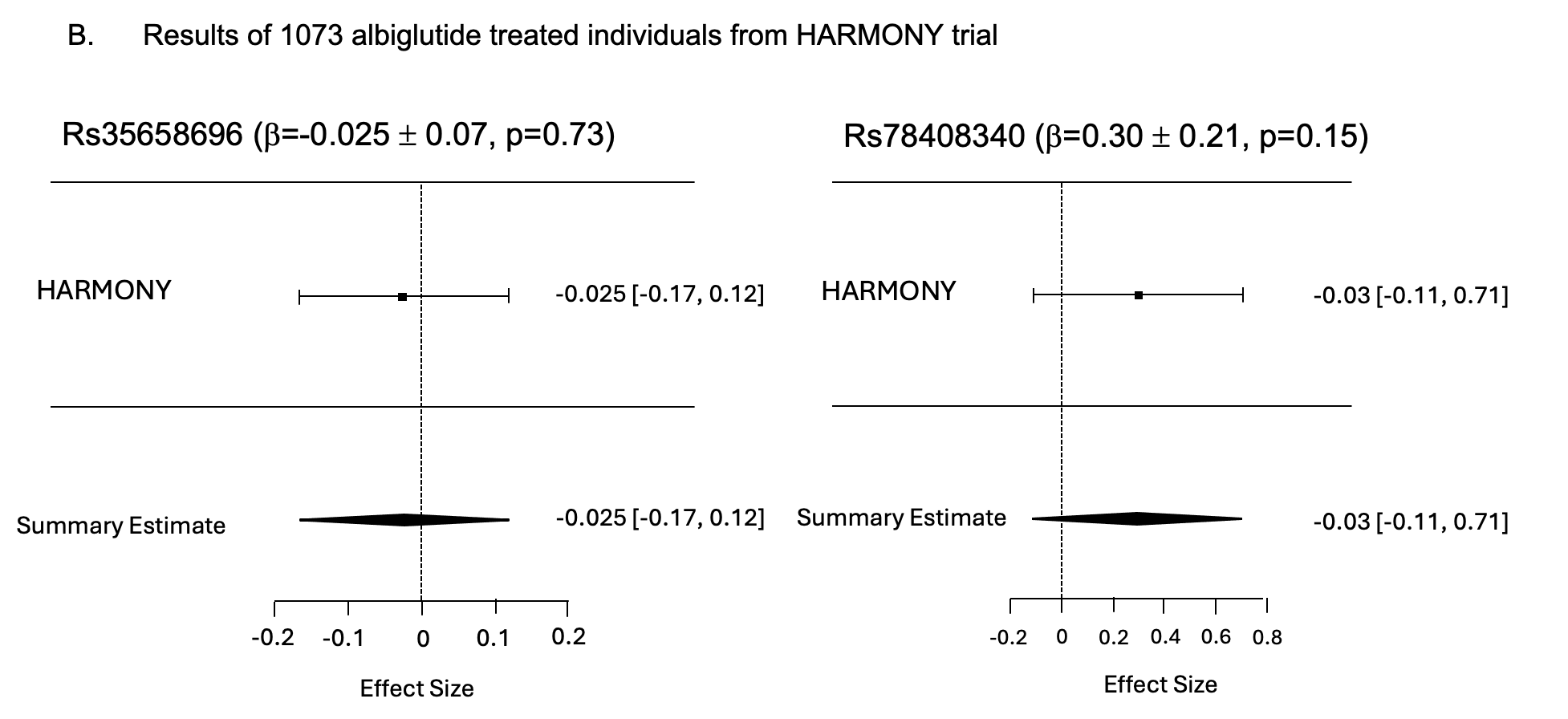
**


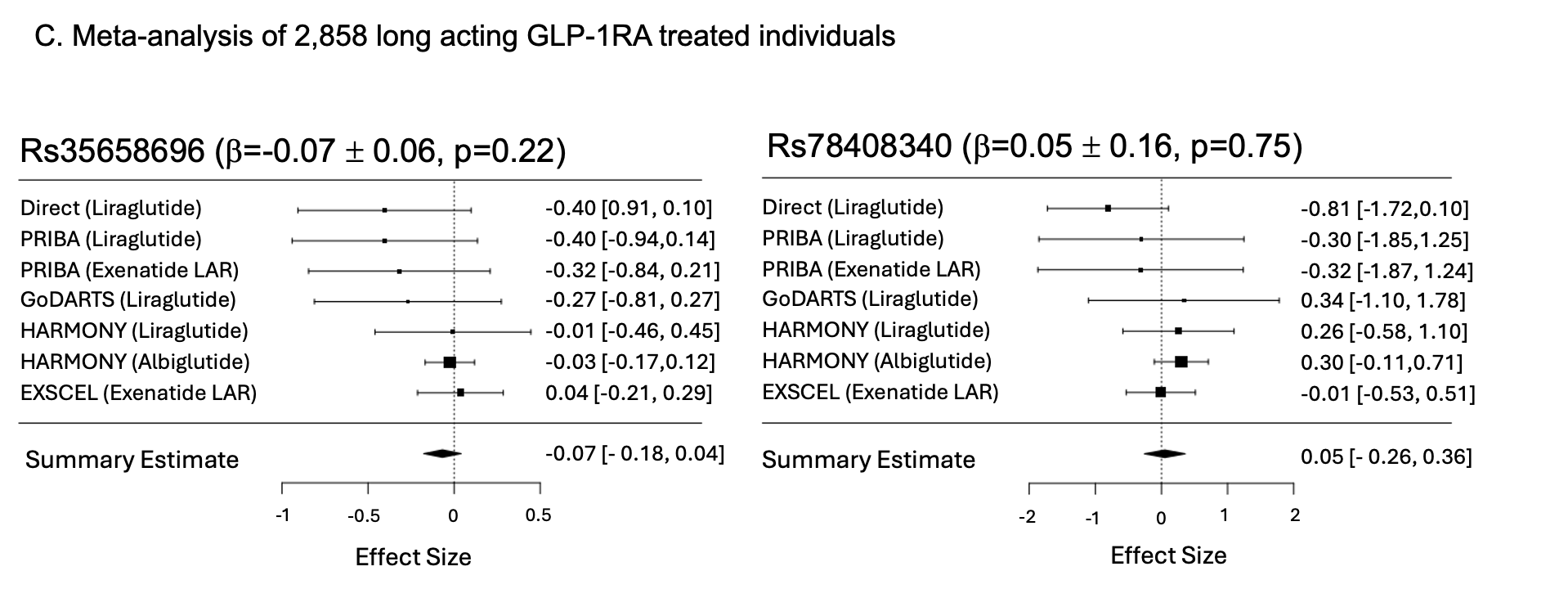


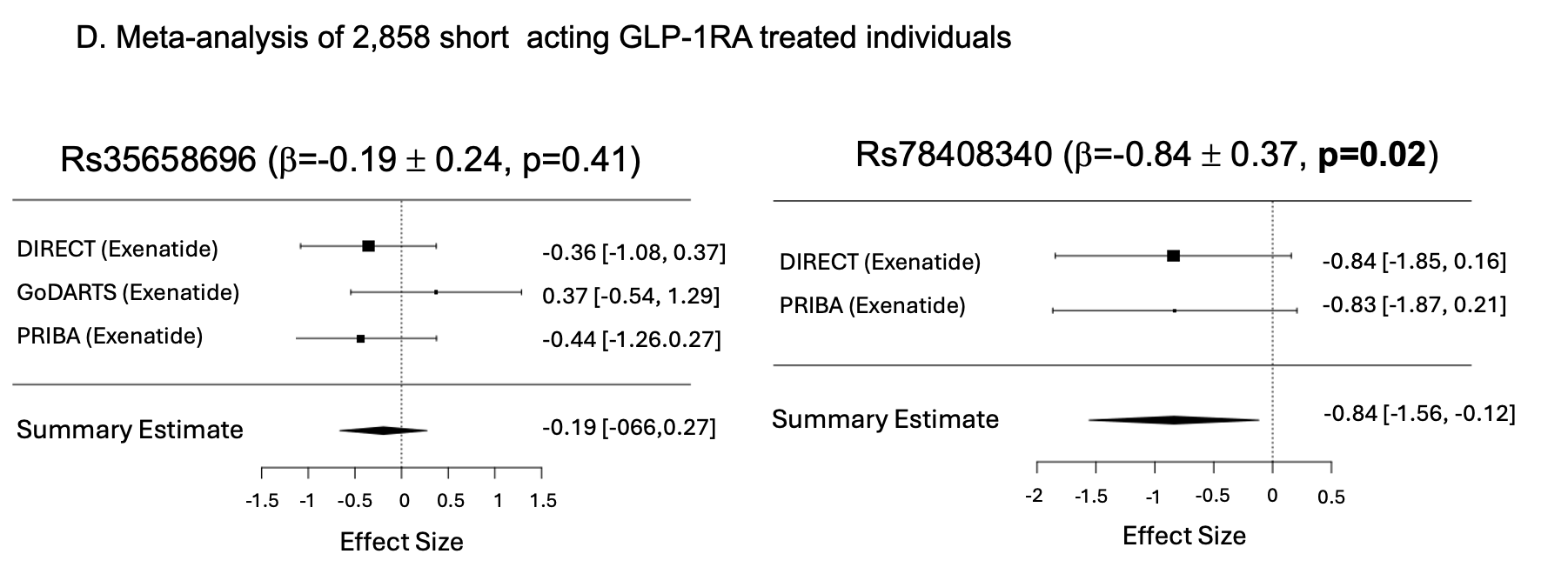


**Figure S8:** **Effect of genotype on weight change for either p.S539W or p.D563G in the albiglutide treated Harmony study**

Figure 8 demonstrates the effect of carrying D536G and S539W on treatment response to GLP-1RA as measured by change in weight in kilograms at 6 months of treatment. Each cohort is displayed separately and the effect size is indicated by the location of a solid box with the 95% CI displayed either side. The line of no effect is indicated by a vertical dotted line. Left of the vertical dotted line demonstrates less weight change, right of the vertical dotted line demonstrates more weight change. The summary estimate of the effect of each allele is displayed below the individual cohort summaries and is indicated by a solid black diamond with the centre of the diamond indicating the summary estimate and the lateral points the 95%CI.

**
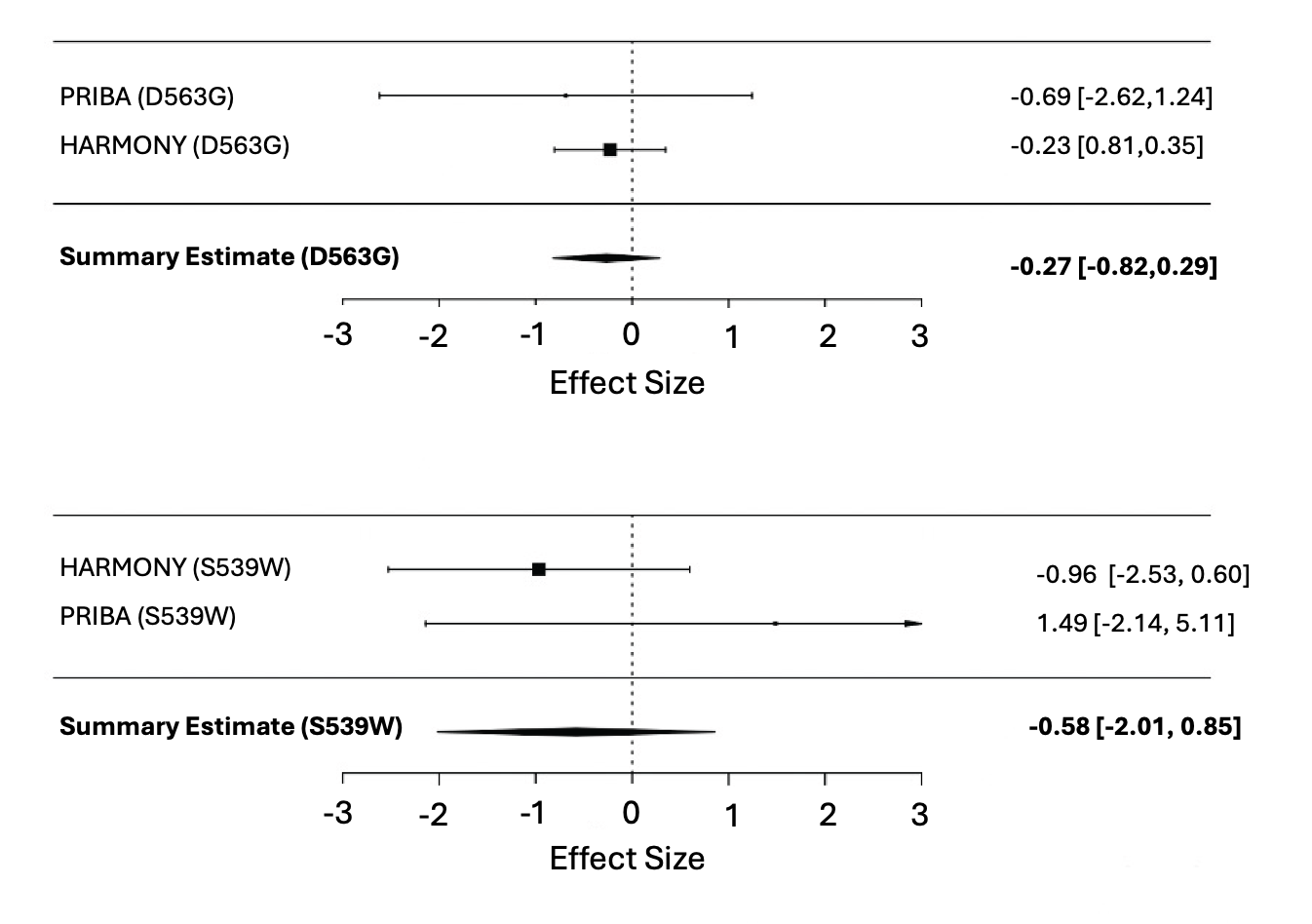
**

**Figure S9:** **Effect of PAM knock-down (KD) on GLP1 stimulation of insulin secretion in human beta cell line (EndoC-βh1) and Glucagon concentration in heterozygous carriers of p.S539W and age, sex and BMI matched controls during oral glucose tolerance test.**

Panel A: EndoC-βH1 cells were transfected with siRNA either control (siControl, grey) or targeted to PAM transcript (siPAM, teal) and stimulated as labelled (n=3 biologically independent experiments, 2-way ANOVA). The GLP-1 concentration used for stimulation is 1nM. Panel B: Glucagon was measured at 9 timepoints during an 75g OGTT. Non-carrier are displayed in blue and heterozygous carriers of p.S539W are displayed in red. There is a significant time by genotype interaction and timepoints 0,15,30 and 240 are significantly different (p<0.05) without correction for multiple testing


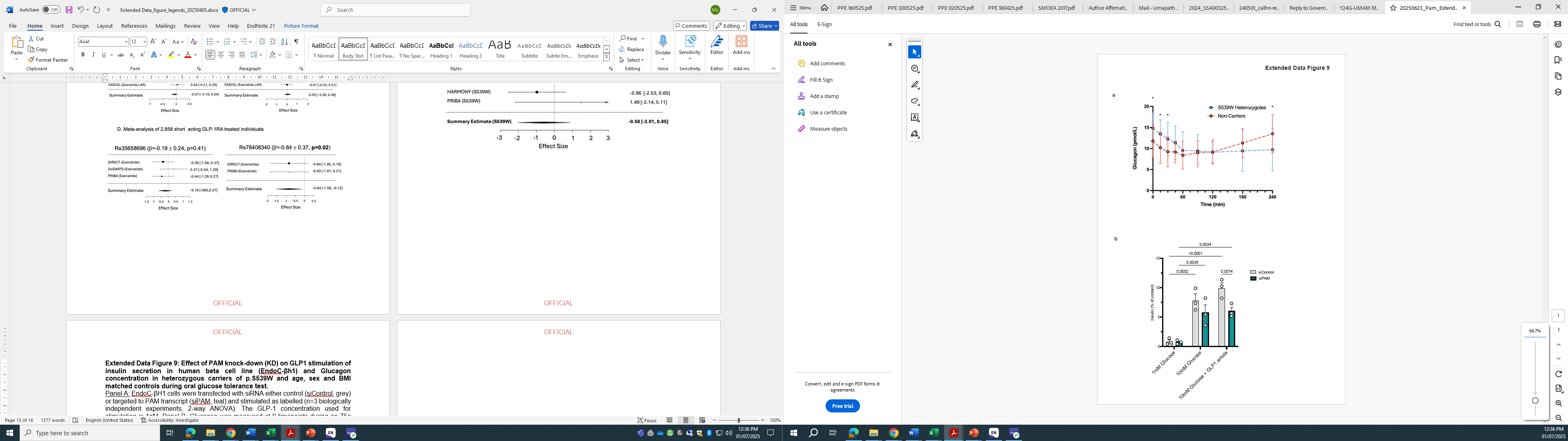

Supplement: Supplementary file 2 — Supplementary Material 2: contains supplementary Figs. 1–9. [file 13073_2026_1630_MOESM2_ESM.docx]
